# Astrocytes display ultrastructural alterations and heterogeneity in the hippocampus of aged APP-PS1 mice and human post-mortem brain samples

**DOI:** 10.1186/s12974-023-02752-7

**Published:** 2023-03-14

**Authors:** Marie-Kim St-Pierre, Micaël Carrier, Fernando González Ibáñez, Mohammadparsa Khakpour, Marie-Josée Wallman, Martin Parent, Marie-Ève Tremblay

**Affiliations:** 1 Axe Neurosciences, Centre de Recherche du CHU de Québec-Université Laval, Québec, QC Canada; 2 Départment de Médecine Moléculaire, Faculté de Médecine, Université Laval, Québec, QC Canada; 3 Division of Medical Sciences, Medical Sciences Building, University of Victoria, Victoria, BC Canada; 4 Département de Psychiatrie et de Neurosciences, Faculté de Médecine, Université Laval, Québec, QC Canada; 5 CERVO Brain Research Center, Quebec City, QC Canada; 6 Department of Biochemistry and Molecular Biology, University of British Columbia, Vancouver, BC Canada; 7 Department of Neurology and Neurosurgery, McGill University, Montreal, QC Canada; 8 Centre for Advanced Materials and Related Technology (CAMTEC), University of Victoria, Victoria, BC Canada; 9 Institute on Aging and Lifelong Health, University of Victoria, Victoria, BC Canada

**Keywords:** Astrocytes, Heterogeneity, Dark astrocytes, Ultrastructure, Aging, Alzheimer’s disease, APP-PS1 mice, Human post-mortem brain samples

## Abstract

The past decade has witnessed increasing evidence for a crucial role played by glial cells, notably astrocytes, in Alzheimer’s disease (AD). To provide novel insights into the roles of astrocytes in the pathophysiology of AD, we performed a quantitative ultrastructural characterization of their intracellular contents and parenchymal interactions in an aged mouse model of AD pathology, as aging is considered the main risk factor for developing AD. We compared 20-month-old APP-PS1 and age-matched C57BL/6J male mice, among the ventral hippocampus CA1 *strata lacunosum-moleculare* and *radiatum*, two hippocampal layers severely affected by AD pathology. Astrocytes in both layers interacted more with synaptic elements and displayed more ultrastructural markers of increased phagolysosomal activity in APP-PS1 *versus* C57BL6/J mice. In addition, we investigated the ultrastructural heterogeneity of astrocytes, describing in the two examined layers a dark astrocytic state that we characterized in terms of distribution, interactions with AD hallmarks, and intracellular contents. This electron-dense astrocytic state, termed dark astrocytes, was observed throughout the hippocampal parenchyma, closely associated with the vasculature, and possessed several ultrastructural markers of cellular stress. A case study exploring the hippocampal head of an aged human post-mortem brain sample also revealed the presence of a similar electron-dense, dark astrocytic state. Overall, our study provides the first ultrastructural quantitative analysis of astrocytes among the hippocampus in aged AD pathology, as well as a thorough characterization of a dark astrocytic state conserved from mouse to human.

**Supplementary Information:**

The online version contains supplementary material available at 10.1186/s12974-023-02752-7.

## Introduction

Alzheimer’s disease (AD), the most common type of dementia, in which aging is a predominant risk factor [1], is associated clinically with progressive brain atrophy, as well as neuronal and synaptic loss, leading over the years to cognitive decline [2–6]. Notable pathological hallmarks of AD include the build-up of intracellular hyperphosphorylated tau forming neurofibrillary tangles (NFTs) and the extracellular accumulation of amyloid beta (Aß) compacting into fibrillar Aß plaques [2]. AD is now considered a brain manifestation of metabolic disorder: signs of AD that start early on during its progression include reduced brain energy metabolism, resulting from alterations in lipid [7–10] and glucose [11] metabolism, as well as amino acids, and other tricarboxylic acid cycle (TCA) metabolites [9, 12], all of which are important to maintain adequate brain energy levels.

A particular feature of AD is the brain region-dependent vulnerability to pathology, starting to affect early on key regions such as the entorhinal cortex and hippocampus [13, 14]; the latter being mainly involved in the regulation of emotions (ventral hippocampus) as well as learning and memory processes (dorsal hippocampus) [15, 16]. The hippocampus, and in particular its *cornu ammonis* (CA)1, have been extensively investigated in the context of AD, due to the drastic atrophy observed [3–5], along with the functional impairment associated with this region throughout the pathogenesis of AD [17, 18]. The CA1 can be further separated into various layers, each defined by distinct functional, structural, and ultrastructural characteristics. For instance, the CA1 pyramidal neurons project their apical dendrites to the *stratum radiatum*, which contains their proximal branches while the *stratum lacunosum-moleculare* contains their distal branches [17]. The *stratum lacunosum-moleculare* also presents numerous large blood vessels [17], the latter being highly vulnerable to AD-related damage most likely due to a reduced blood flow at baseline compared to other brain regions [19].

Astrocytes, representing roughly 20–40% of all glial cells in the cerebral gray matter [20, 21], first originate during embryonic development from progenitor cells or radial glia which then mature throughout early postnatal development into astrocytes [22]. Defined morphologically by their star-like appearance given their numerous and complex processes [23], astrocytes are characterized, at the ultrastructural level, by their angular and thin processes often interacting with synaptic elements, their intermediate filaments, and the accumulation of glycogen granules [24]. Astrocytes can help protect brain homeostasis through the clearance of parenchymal metabolic waste through the glymphatic system [25–28], the regulation of cerebral blood flow, the maintenance of the blood–brain barrier [29–32], and are highly involved in synaptic activity and plasticity (synaptogenesis, maintenance, maturation, and elimination) [33–37]. Known for their key role in neuronal metabolic support, astrocytes can help maintain brain functions in numerous ways; for instance, by transforming the exocytotoxic glutamate released by post-synaptic dendritic spines into glutamine, which can be recycled back by synaptic elements [38, 39]. In addition, astrocytic glycogen plays a crucial role in the metabolic neuronal support as the current hypothesis suggests that astrocytes can break down this carbohydrate storage into lactate, which can be shuttled to neurons for their energetic needs [40, 41].

The last decades have provided increasing evidence that glial cells, including astrocytes, are critical players in the pathogenesis of AD. This emerging role of astrocytes in AD is corroborated notably by genome-wide association studies highlighting several astrocytic gene variants connected with a higher risk of developing late-onset AD in humans [42, 43]. While the functions of astrocytes in the pathogenesis of AD remain to be fully demystified, studies depleting astrocytes (using transgenic or pharmacological strategies) to investigate their role in AD pathogenesis are pointing toward beneficial outcomes [44]. In particular, increased Aß levels were measured upon astrocytic ablation in two models of AD pathology: organotypic brain cultures from postnatal day 7 5xFAD mice and hippocampal sections from 9-month-old APP23/glial fibrillary acidic protein (GFAP)-thymidine kinase mice [45, 46], highlighting a potential role of astrocytes in the clearance of Aß. Astrocytes near Aß plaques were also shown to release neprilysin, an enzyme capable of degrading Aß [47], via protein kinase A and C [48], as well as insulin [49], a hormone crucial for the regulation of glucose metabolism [50]. At the ultrastructural level, astrocytic processes were shown to penetrate inside the Aß plaque core [51], suggested to be associated with plaque fragmentation to help with its degradation in human post-mortem brain samples of patients with AD [52]. Astrocytes were also confirmed to engulf dystrophic neurites, often found accumulated nearby Aß plaques in 6- and 12-month-old APP-PS1 mice, a model of AD pathology [53]. A more recent ultrastructural investigation in aged human post-mortem brain samples of individuals with AD further demonstrated that the astrocytic density near Aß plaques did not correlate to plaque size, and hypothesized that their close interaction with the plaque microenvironment could be due to neuritic damage rather than the Aß plaque itself [54].

Studies using single-cell and -nucleus RNA sequencing further demonstrated the highly heterogeneous nature of astrocytes in response to AD pathology, with a myriad of transcriptomic signatures reported such as the disease-associated astrocytes [55–57]. This signature was uncovered in 1.5–2, 4–5, 7–8, 10, 13–14 and 20-month-old male and female 5xFAD mice and presented an upregulation of specific genes such as *apoe* and *clu*, both involved in Aß clearance [55]. However, the phenotypic alterations and heterogeneity of astrocytes in AD have not been examined yet at the ultrastructural level using electron microscopy, an approach which provides in-depth knowledge at the nanoscale on the structure of organelles and the cellular interactions among the parenchyma [56]. Understanding the structural alterations of astrocytic organelles, their intracellular contents (notably the nature and quantity of phagosomes), as well as their interaction with AD hallmarks will aid in our understanding of their roles in AD pathology. In addition, as morphological and transcriptomic studies have reported a plethora of astrocytic signatures with varying functions, investigating the heterogeneity of astrocytes on an ultrastructural level will complement previous studies and help mend the gap in unraveling in situ the diverse responses of astrocytes to AD pathology.

This study aimed to provide quantitative data on the ultrastructure of astrocytes and assess qualitatively their heterogeneity among the ventral hippocampus CA1 *strata lacunosum-moleculare* and *radiatum*, layers highly affected by AD pathology [17, 58]. APP-PS1 and control C57BL/6J male mice were examined at 20 months of age to focus on aged AD pathology specifically. Astrocytes from the two examined layers showed increased interactions with synaptic elements (dendritic spines and axon terminals), along with an increased phagolysosomal pathway activity (more phagosomes and/or mature lysosomes within their cytoplasm). In addition, we uncovered electron-dense, dark astrocytic cells for the first time in aging and AD pathology, possessing ultrastructural features of astrocytes and markers of cellular stress, similar to the dark microglia [59, 60] and similar to dark astrocytic states observed in human post-mortem brain samples of brain injury [61–63] and brain tumors [62, 64] resected following surgery, in rat models of brain injuries (concussive and compressive head injuries [65] and electroshock [66], as well as in spinal cord cultures of embryonic mice [67]. These dark glial cells were positive for the ‘reactive’ astrocytic marker GFAP [68] and were observed throughout the parenchyma often in juxtaposition with large blood capillaries. Moreover, our observations highlight the presence of dark astrocytes in the hippocampal head of an aged human post-mortem brain sample, examined as a case study, similarly to the dark astrocytes previously observed in the parietal cortex of patients with traumatic brain injury and brain tumors [62, 63]. These findings confirmed the conservation across species of dark astrocytes as these cells were encountered in human post-mortem brain samples, thus showcasing similarities in the astrocytic ultrastructural features observed upon aging between mouse and human.

## Methods

### Animal housing, euthanasia, and perfusion with aldehydes

All experiments were performed according to the guidelines of the Institutional Animal Ethics committees, the Canadian Council on Animal Care, as well as the Animal Care Committee of Université Laval. C57BL/6J and age-matched APP^Swe^-PS1Δe9 male mice on a C57BL/6J background [69] (No. 34832-JAX, Jackson Laboratory, Maine, USA) at 3–4, and 20 months of age (*n* = 3–4), were housed under a 12 h light–dark cycle at 22–25 °C with free access to food and water. All experiments were performed on males, for this first study on the topic, considering that previous studies investigated glial heterogeneity in 14- and 20-month-old C57BL/6J and APP-PS1 mice used males [70, 71]. Mice were injected with 10 g/kg Methoxy-X04 (Tocris Biosciences, cat# 4920, Bristol, United Kingdom) 24 h prior to their euthanasia to visualize fibrillar Aß plaques at the light microscopy level [72]. Mice were injected intraperitoneally with sodium pentobarbital (80 mg/kg), then perfused transcardially with 3.5% acrolein [diluted in phosphate buffer (PB): 100 mM at pH 7.4] and 4% paraformaldehyde [PFA, diluted in phosphate-buffered saline (PBS): 50 mM at pH 7.4], followed by a 2-h post-fixation in 4% PFA. Coronal brain sections were cut using a vibratome (Leica VT1000S) at 50 µm of thickness and kept in a cryoprotectant solution [20% glycerol (v/v), 20% (v/v) ethylene glycol in PBS] at − 20 °C until further processing.

### Processing of human post-mortem brain samples

As a case study, sections from a human brain (female, 81 years old; 18 h post-mortem delay, cause of death: asphyxia) were obtained from the CERVO Brain Research Center (QC, Canada). Collecting, storage and handling procedures were approved by the Ethics Committee of the Institut Universitaire en Santé Mentale de Québec and Université Laval. Written and informed consent was obtained for the use of human post-mortem brain tissues and all the experiments were performed in line with the Code of Ethics of the World Medical Association. The brain was first separated in halves trough the midline and hemibrains were cut coronally in 2-cm-thick blocks. They were then fixed in 4% PFA for 3 days at 4 °C before being stored in 15% sucrose and 0.1% sodium azide at 4 °C until further processing. The hippocampal head region of the right hemibrain was then cut using a vibratome (VT1000s) to obtain 50-µm-thick coronal sections which were kept at − 20 °C in a cryoprotectant solution until further processing, in preparation for scanning electron microscopy (SEM) experiments.

### Processing of mouse samples for anti-GFAP immunohistochemistry

Brain sections containing the ventral hippocampus CA1 from 20-month-old APP-PS1 male mice (Bregma 2.92 to 3.64 mm [73]) were selected for further processing. Selected sections were quenched with 0.3% H_2_O_2_ (Fisher Scientific, Ottawa, lot# 202762) in PBS for 5 min. Afterward, the sections were incubated in 0.1% NaBH_4_ in PBS for 30 min followed by 3 washes of 10 min in PBS. Brain sections were then incubated in a blocking buffer solution containing 5% normal goat serum (Jackson ImmunoResearch Labs, Baltimore, USA cat# 005-000-121), 5% bovine albumin serum (Sigma-Aldrich, Oakville, cat# 9048-46-8,), and 0.01% Triton X-100 in PBS for 1 h at room temperature (RT). They were then incubated overnight in a blocking buffer solution with the primary rabbit polyclonal anti-GFAP antibody (1:5000; Abcam, Cambridge, MA, USA, Ab7260) at 4 °C. The following day, the sections were washed in 0.01% PBS-Triton (PBS-T) and incubated with a biotinylated goat anti-rabbit polyclonal secondary antibody (1:300; Jackson ImmunoResearch, Baltimore, USA, cat# 111-066-046) in Tris-buffered saline (TBS; 50 mM, pH 7.4) for 2 h at RT. Afterward, the sections were washed in PBS-T and incubated for 1 h at RT in an avidin–biotin complex solution (ABC; 1:100; Vector Laboratories, Newark, USA, cat# PK-6100) in TBS. The staining was revealed with a solution containing 0.05% 3,3′-diaminobenzidine (DAB; Millipore Sigma, Oakville, USA, cat# D5905-50TAB) and 0.015% H_2_O_2_ diluted in Tris buffer (TB; 0.05 M, pH 8.0). The samples were washed 3 times in PBS and then further processed with unstained sections for SEM.

### Preparation of mouse and human samples for SEM

Mouse brain sections containing the ventral hippocampus CA1 (Bregma 2.92 to 3.64 mm [73]) from 3–4- and 20-month-old C57BL/6J mice and age-matched APP-PS1 mice, both unstained for quantitative analysis and stained for GFAP to confirm the astrocytic identity, were selected for SEM processing. As a case study, post-mortem human brain samples containing the hippocampal head from an aged individual were also processed for SEM. Selected sections were first washed with PB, then incubated for 1 h in a PB solution containing equal volumes of 3% potassium ferrocyanide (Sigma-Aldrich, Ontario, Canada, cat# P9387) and 4% osmium tetroxide (EMS, Pennsylvania, USA, cat# 19190). The brain tissues were next incubated for 20 min in a filtered and heated 1% thiocarbohydrazide solution (diluted in double-distilled water; Sigma-Aldrich, Ontario, Canada, cat# 223220) and for 30 min in 2% aqueous osmium tetroxide. The samples were dehydrated in increasing concentrations of ethanol for 10 min each (2 × 35%, 1 × 50%, 1 × 70%, 1 × 80%, 1 × 90% 3 × 100%) followed by 3 washes of 10 min in propylene oxide (Sigma-Aldrich, Ontario, Canada, #cat 110205-18L-C). The dehydrated tissues were embedded overnight in Durcupan resin (20 g component A, 20 g component B, 0.6 g component C, 0.4 g component D; Sigma Canada, Toronto, cat# 44,610) and flat-embedded between fluoropolymer films (ACLAR^®^, Pennsylvania, USA, Electron Microscopy Sciences, cat# 50425–25). Resin-embedded sections between films were kept in the oven for 5 days at 55 °C to allow the resin to polymerize.

Regions of interest (containing the hippocampal head for post-mortem human brain and the ventral hippocampus CA1 *strata lacunosum-moleculare* and *radiatum* for mouse brain samples) were excised from the resin-embedded sections and glued onto resin blocks for ultramicrotomy. Using a Leica ARTOS 3D ultramicrotome, 73-nm-thick sections were cut with multiple levels obtained from each block (2–6 levels, ~ 6 µm apart) to obtain sufficient images of astrocytes for quantitative ultrastructural analysis. The ultrathin sections were placed on silicon wafers for SEM imaging, performed using a Zeiss Crossbeam 540 microscope. Images from mouse samples were first acquired at 25 nm per pixel for the density and distribution analysis of astrocytes [70]. All samples were imaged at a resolution of 5 nm per pixel for the ultrastructural analysis of typical astrocytes and characterization of dark astrocytes. GFAP-positive (+) typical astrocytes and dark astrocytes were further imaged with a Zeiss Crossbeam 350 scanning electron microscope using SmartSEM software (Fibics). GFAP + dark and typical astrocytic cell bodies were imaged at a resolution of 5 nm and 1 nm per pixel and exported as TIFF files using the Zeiss ATLAS Engine 5 software (Fibics).

### Density and distribution analysis of astrocytic states in mouse samples

Parenchymal images (2–6 levels, ~ 6 µm apart) from the ventral hippocampus CA1 *stratum lacunosum-moleculare* from 4 animals per group were blinded to the genotype and age, then analyzed to investigate the density and distribution of astrocytic states. A distinction was made between dark and non-dark astrocytes (referred to as typical astrocytes in this manuscript) based on our ultrastructural observations. The density of typical and dark astrocytes in APP-PS1 *vs* C57BL/6J mice was determined, together with the ratio of dark astrocytes over all astrocytes imaged in each genotype using the 25 nm per pixel resolution images. In addition, we investigated the distribution of astrocytes based on their association with the vasculature or parenchyma (with or without any direct contact with the basement membrane of blood vessels, respectively). Typical astrocytes were positively identified based on their electron-lucent cyto- and nucleoplasm, granular nuclear pattern, angular processes interacting with parenchymal elements, as well as the presence of intermediate filaments [24, 60, 74]. A dark astrocytic state, termed dark astrocytes, was also identified based on the similar ultrastructural defining features of typical astrocytes, such as the angular processes and granular pattern of the nucleus, as well as presence of intermediate filaments, and previous EM observations made in organotypic cultures of spinal cord from embryonic mice [67], rat models of brain injury (compressive head injury, concussive head injury), pentylenetetrazole and kainic acid treatment [65], as well as electroshock [66]. The dark astrocytes that we observed often possessed a high accumulation of glycogen granules, ultrastructural markers of cellular stress such as the dilation of the endoplasmic reticulum (ER) and Golgi apparatus cisternae, a partial to total loss of their nuclear heterochromatin pattern, and an electron-dense cyto- and nucleoplasm [65–67]. Similar ultrastructural features were previously described in dark neurons [75–79] and dark microglia [59, 60, 70, 80], particularly the loss of nuclear heterochromatin pattern, electron-dense cytoplasm and nucleoplasm, and markers of cellular stress [60, 71, 72, 80, 81]. The ultrastructural density analysis protocol we performed for typical and dark astrocytes is based on previously published ultrastructural work examining microglia [60, 70].

### Ultrastructural analysis of typical astrocytes in mouse samples

For the ultrastructural analysis of typical and dark astrocytes, quantitative and qualitative, respectively, SEM images captured with a resolution of 5 nm per pixel were used. This analysis was conducted in the ventral hippocampus CA1 *strata lacunosum-moleculare* and *radiatum* from 20-month-old C57BL/6J and APP-PS1 mice. In each genotype (*n* = 3 animals/group) and localization (near *vs* far Aß plaques/dystrophic neurites in the case of the *stratum lacunosum-moleculare*), pictures of 31–38 astrocytes were acquired. Of note, in the *stratum radiatum,* we investigated astrocytes far from Aß plaques/dystrophic neurites only as little to no plaques were observed in this layer in our ultrathin samples. All the images were blinded to the experimental conditions. In the *stratum lacunosum*-*moleculare*, we analyzed a total of 102 astrocytic cell bodies per group, a sample size sufficient to obtain statistical power based on the software G*Power V3.1 (effect size of 0.4; power of 0.95 estimated at 102 astrocytes). In the *stratum radiatum*, we analyzed a total of 59 astrocytic cell bodies per genotype to obtain sufficient statistical power (effect size of 0.9; power of 0.9 estimated at 60 astrocytes) [70]. These effect sizes were previously used to assess the ultrastructural heterogeneity of other glial cells, such as microglia [70, 82]. As we wanted to examine possible glycogen granules within the astrocytic cytoplasm as well as the electron density of their nucleoplasm and cytoplasm in our analysis of their ultrastructure, we did not perform immunostaining which could have masked these features. While no quantitative ultrastructural analysis of astrocytes had been performed yet, the identification and analysis of microglial intracellular contents and parenchymal interactions were previously described in detail [24, 60, 80, 83]. In the current study, the parenchymal interactions of astrocytes with myelinated axons, axon terminals, dendritic spines, and both elements of excitatory synapses were assessed. Myelinated axons were characterized by electron-dense sheaths surrounding the axonal cytoplasm [84]. Axon terminals contained several synaptic vesicles and sometimes juxtaposed dendritic spines recognized by their post-synaptic density [24, 74, 83]. Axon terminals that were or were not in direct contact with one or more dendritic spines were analyzed. Direct contacts with axon terminals, dendritic spines, and both elements of the same excitatory synapse were counted.

Immature (primary, secondary) and mature (tertiary) lysosomes were identified by their homogenous or heterogeneous appearance, respectively [60, 71, 74]. The presence of phagosomes, both fully and partially digested, was often recognized among tertiary lysosomes, alongside large lipid droplets [60, 71]. The latter possessed a homogenous interior (either electron-lucent or dense) and an electron-dense outline [24, 60, 74, 80]. Fully or partially digested phagosomes were characterized by a defined membrane delineating a circular or oval shape, electron-lucent interior with (partially digested) or without (fully digested) cellular content [60, 70]. Likewise, autophagosomes possessed a circular double membrane, with an electron-lucent appearance in between the latter, and an interior with the same electron density as the cell’s cytoplasm [24, 60, 74].

Ultrastructural markers of cellular stress were assessed including the presence of altered mitochondria, as well as dilated ER and Golgi apparatus cisternae. The width of ER and Golgi cisternae, together with the length of mitochondria were measured using ImageJ. ER were identified by their long and narrow stretches, while dilation of their cisternae was positively confirmed when swollen electron-lucent pockets measured at least 100 nm in diameter [59, 60, 71, 85]. Similarly, Golgi apparatuses, characterized by their beehive shape, were considered to have dilated cisternae when displaying swollen electron-lucent pockets larger than 100 nm [60, 70]. Mitochondria were defined as electron-dense double-membraned organelles possessing several cristae [60]. Mitochondria were considered to be ultrastructurally altered when their outer and/or inner membranes were degraded, if their cristae were deteriorated resulting in electron-lucent space, or if they had a “holy shape” indicative of mitochondria wrapping around themselves, a feature associated with impaired mitochondrial membrane potential and structural alterations thought to be associated with oxidative stress [60, 80, 86]. Mitochondria were also defined as elongated if their length measured over 1 µm [85]. The mitochondrial network, defined by the cytoplasmic area occupied by the mitochondrial area, was assessed [87]. Each mitochondrion was traced using the “freehand tool” in Image J, and the sum of all mitochondrial area was divided by the area of the cytoplasm to obtain the mitochondrial network [87]. The presence of glycogen granules, recognized as 22–40 nm electron-dense puncta contained within the astrocytic cytoplasm, was identified [88]. Lastly, nuclear indentations, a phenomenon associated with cell morphology remodeling [89] and observed as hollowed-out portions of the nucleus [90] were noted.

Shape descriptors of astrocytes, i.e., area, perimeter, solidity, aspect ratio (AR), and circularity, were further measured using the software Image J. AR and circularity provide information on the elongation of the cells (AR is the ratio of height over width; circularity is 4π times the area over the perimeter squared) [84, 91]. The closer the value of the circularity to 0, the more elongated the cell body is [70, 71, 84, 92]. Solidity, a measurement of irregularity, is defined by the area of the cell body over the convex area (the closer the value to 0, the more irregular the shape is) [84, 91].

### Qualitative analysis of typical and dark astrocytes in human samples

The presence of typical and dark astrocytes in the hippocampal head of human post-mortem brain samples from an aged individual (female; post-mortem delay of 18 h; cause of death: asphyxia) was investigated as a case study, using similar identifying features for mouse astrocytes, and others described for astrocytes in the parietal and frontal cortical regions of human post-mortem samples resected following surgery of brain injury investigating qualitatively their ultrastructure [61]. In brief, astrocytes were positively identified by their angular processes protruding from the cell body, granular nucleus, and presence of intermediate filaments [24, 60–62, 74]. Dark astrocytes possessed similar ultrastructural characteristics alongside an electron-dense cytoplasm and nucleoplasm, as well as markers of cellular stress (e.g., dilated ER and altered mitochondria). Previous studies investigating human post-mortem cerebral cortex samples with brain injuries or cerebellar samples with hemangioblastoma resected following surgery identified similar dark astrocytes, which were termed “dark hypertrophic astrocytes” [61–63] and “dark astrocytes”, respectively [64]. These dark astrocytes were previously described as electron-dense cells with swollen mitochondria, abundant glycogen granules, and dilated ER cisternae [61–64]. The intracellular contents (e.g., mitochondria, fully and partially digested phagosomes, dilated ER, lysosomes) and parenchymal interactions (e.g., axon terminals, dendritic spines, myelinated axons) of these dark astrocytes were identified for the first time during aging among the hippocampal head based on similar criteria as in the mouse samples and those described in the aforementioned studies [61, 62, 64, 65, 67].

### Statistical analysis

Statistical analysis was performed using the software Prism 9 (v.9.2.0 GraphPad). For all quantitative data obtained (ultrastructure and cellular density in mice), the normality of the data distribution was first assessed using a Shapiro–Wilk test. The ultrastructural data of typical astrocytes in the *stratum radiatum* of C57BL/6J *vs* APP-PS1 mice were compared with a Mann–Whitney non-parametric test. The ultrastructural data of typical astrocytes in the *stratum lacunosum* of C57BL/6J *vs* APP-PS1 mice (far *vs* near Aß plaques/dystrophic neurites) were analyzed with a Kruskal–Wallis one-way ANOVA followed by a Dunn’s post hoc test. The density data of dark and typical astrocytes in the *stratum lacunosum-moleculare* of APP-PS1 mice *vs* C57BL/6J mice passed normality and were analyzed with a Welsh *t*-test. Data are expressed as mean ± standard error of mean (SEM). The sample size (*n*) refers to individual animals for the density analysis and individual astrocytes for ultrastructural analysis as performed in previous EM studies studying other glial cell types such as microglia to account for the ultrastructural heterogeneity between individual cells [70, 71, 84, 93–97]. Statistically significant differences are reported as *p < 0.05, **p < 0.01, ***p < 0.001, and ****p < 0.0001.

## Results

### Typical astrocytes in the hippocampal CA1 *stratum radiatum* of aged APP-PS1 *vs* age-matched C57BL/6J mice exhibit altered parenchymal interactions and intracellular contents

The ventral (or anterior) hippocampus CA1 displays severe atrophy [3, 5, 98–101], as well as astrocytic morphological and molecular alterations [39, 102, 103], in samples from mouse models of AD pathology and patients with AD. We thus analyzed the ultrastructural features of astrocytes in these two layers of the ventral hippocampus CA1. This region is of particular interest as previous studies which were conducted in middle-aged and aged APP-PS1 mice revealed ultrastructural alterations together with an increased heterogeneity of microglia, another glial cell type highly affected by AD pathology and known to play a role in its pathogenesis [59, 70]. Across the study, 20-month-old APP-PS1 were compared with age-matched C57BL/6J male mice. We first started our ultrastructural investigation with the analysis of typical astrocytes in the *stratum radiatum*. We focused on areas located far from Aß plaques/dystrophic neurites (designated as ‘Far AD’) to have a sufficient sample size for this analysis, as little to no plaques were observed in this hippocampal layer among our samples.

**Fig. 1:**
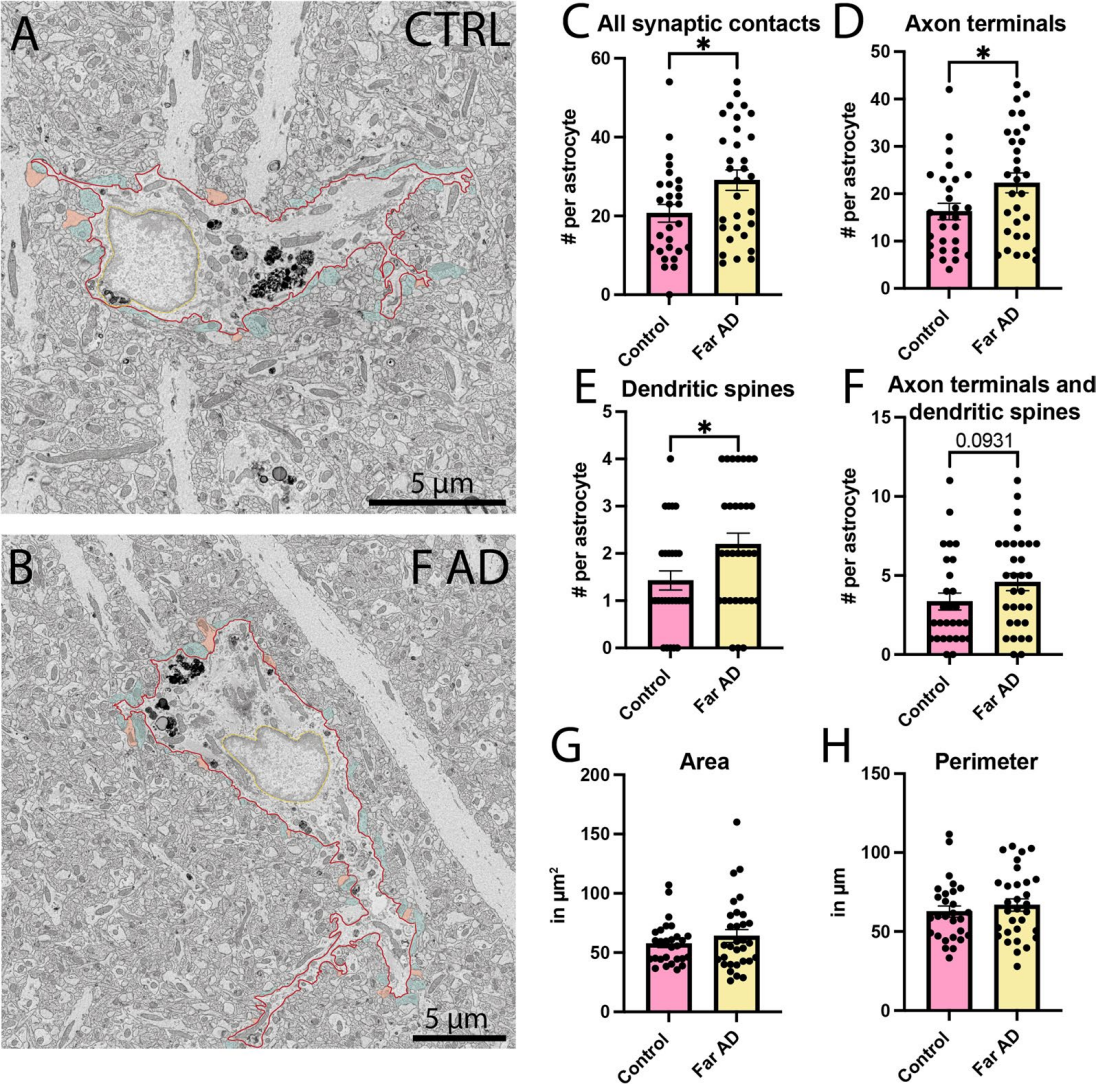
Parenchymal interactions of typical astrocytes in the *stratum radiatum*. Representative 5 nm per pixel of resolution scanning electron microscopy images acquired in the ventral hippocampus CA1 *stratum radiatum* of 20-month-old APP-PS1 (far from Aß plaques/dystrophic neurites) and age-matched C57BL/6J male mice (**A**, **B**). Quantitative graphs represent the number of direct contacts with **C** all synaptic elements, **D** axon terminals, **E** dendritic spines, and **F** both elements of the same synapse (axon terminals and dendritic spines). In **G** and **H**, the graphs represent, respectively, the area and perimeter of the astrocytic cell body. Data are shown as individual dots and are expressed as mean ± S.E.M. *p < 0.05, using a non-parametric Mann–Whitney test. Statistical tests were performed on *n* = 8–11 astrocytes per animal in *N* = 3 mice/group, for a total of 59 cell bodies analyzed. red outline = plasma membrane, yellow outline = nuclear membrane, orange pseudo-coloring = dendritic spine, blue pseudo-coloring = axon terminals

In the *stratum radiatum* (Fig. 1A, B), we observed a significant increase in the direct contacts between typical astrocytes and all synaptic elements in the APP-PS1 mice compared to age-matched C57BL/6J controls (Control 20.68 ± 2.244 contacts per astrocyte *vs* Far AD 29.06 ± 2.587 contacts per astrocyte, *p* = 0.0250) (Fig. 1C). When we further investigated which part of the synapses was contacted by astrocytes, we found increased interactions of astrocytes from APP-PS1 mice with axon terminals (Control 16.25 ± 1.729 contacts per astrocyte *vs* Far AD 22.29 ± 2.089 contacts per astrocyte, *p* = 0.0447) and dendritic spines (Control 1.429 ± 0.2020 contacts per astrocyte *vs* Far AD 2.194 ± 0.2384 contacts per astrocyte, *p* = 0.0243), and a tendency for both elements of a same excitatory synapse to be contacted (Control 3.357 ± 0.5400 contacts per astrocyte *vs* Far AD 4.581 ± 0.5344 contacts per astrocyte, *p* = 0.0931) (Fig. 1D–F). We confirmed that this increased structural relationship with synaptic elements was not due to a change in either the astrocytic area (Control 57.65 ± 3.380 µm^2^ *vs* Far AD 64.01 ± 5.421 µm^2^, *p* = 0.7574) or perimeter (Control 62.65 ± 3.603 µm *vs* Far AD 66.83 ± 3.841 µm, *p* = 0.4196) (Fig. 1G–H). These results highlight the preferential contacts with synapses made by astrocytes in the *stratum radiatum* of APP-PS1 mice compared to age-matched C57BL/6J controls.

**Fig. 2:**
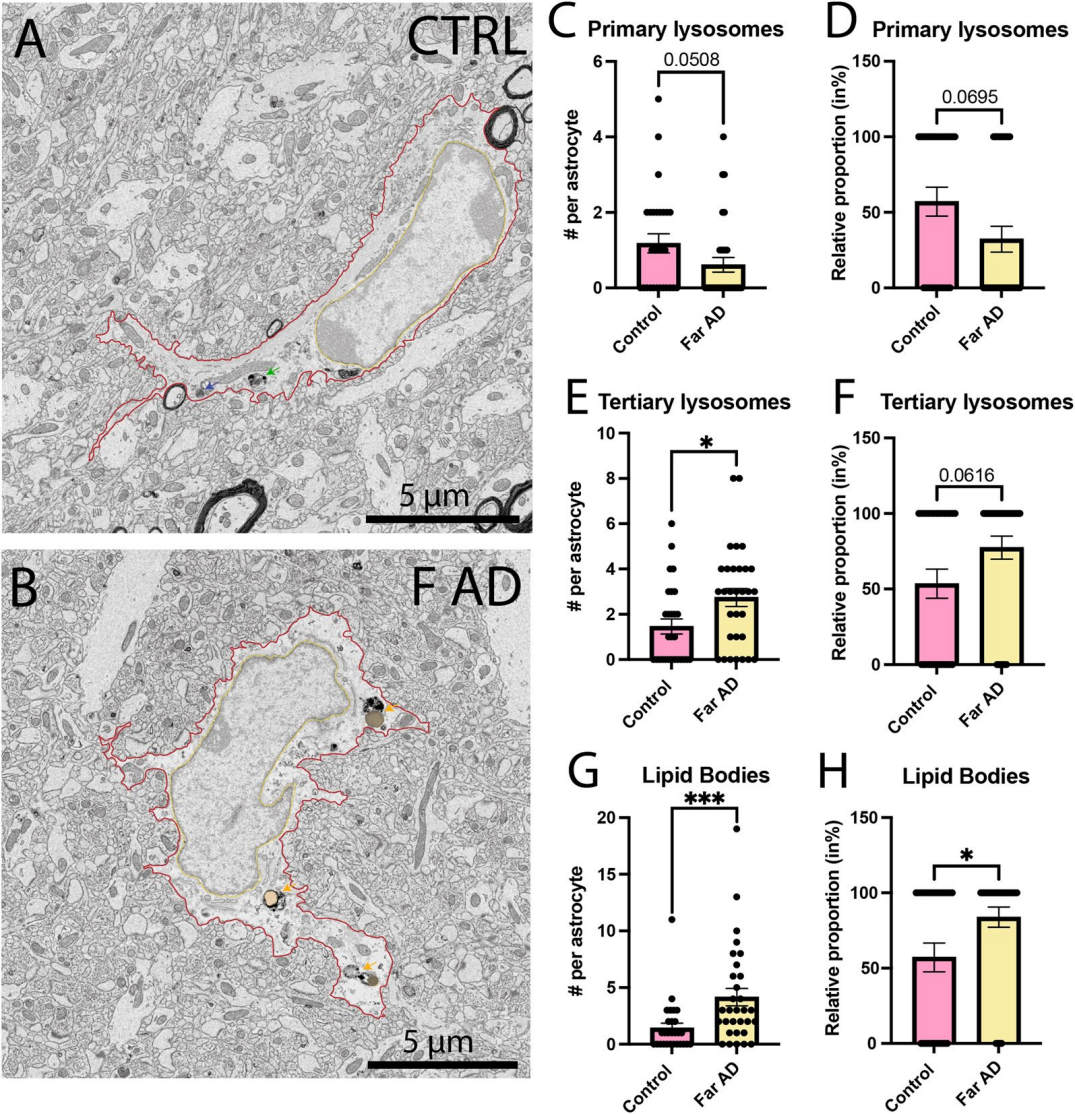
Intracellular contents of typical astrocytes in the *stratum radiatum*. Representative 5 nm per pixel of resolution scanning electron microscopy images acquired in the ventral hippocampus CA1 *stratum radiatum* of 20-month-old APP-PS1 (far from Aß plaques/dystrophic neurites) and age-matched C57BL/6J male mice (**A**, **B**). Quantitative graphs representing the number of primary lysosomes (**C**), tertiary lysosomes (**E**), and lipid bodies (**G**) are provided. Quantitative graphs represent the relative proportion of cells positive for primary lysosomes (**D**), tertiary lysosomes (**F**), and lipid bodies (**H**). Data are shown as individual dots and are expressed as mean ± S.E.M. *p < 0.05, ***p < 0.001, using a non-parametric Mann–Whitney test. Statistical tests were performed on *n* = 8–11 astrocytes per animal in *N* = 3 mice/group, for a total of 59 cell bodies analyzed. Red outline = plasma membrane, yellow outline = nuclear membrane, blue arrow = primary lysosomes, green arrow = secondary lysosomes, orange arrow = tertiary lysosomes, orange pseudo-coloring = lipid bodies

Moreover, in the *stratum radiatum* (Fig. 2A, B), intracellular investigation of astrocytes further revealed a tendency for a decreased presence of primary lysosomes in APP-PS1 mice compared to C57BL/6J controls (Control 1.179 ± 0.2523 primary lysosomes per astrocyte *vs* Far AD 0.6129 ± 0.1950 primary lysosomes per astrocyte, *p* = 0.0508) (Fig. 2C), while the APP-PS1 mice exhibited an increased number of tertiary lysosomes (Control 1.464 ± 0.3313 tertiary lysosomes per astrocyte *vs* Far AD 2.742 ± 0.3934 tertiary lysosomes per astrocyte, *p* = 0.0163) (Fig. 2E). This finding suggests a shift in the phagolysosomal pathway, more precisely an increased maturation of lysosomes resulting in more tertiary lysosomes and less primary lysosomes in the APP-PS1 mice. We observed similar tendencies in the relative percentage of astrocytes (cells positive for the presence of the organelle analyzed) for both primary lysosomes (Control 57.14 ± 9.524% of astrocytes *vs* Far AD 32.36 ± 8.535% of astrocytes, *p* = 0.0695) in the C57BL/6J control mice and tertiary lysosomes (Control 53.57 ± 9.598% of astrocytes *vs* Far AD 77.42 ± 7.634% of astrocytes, *p* = 0.0616) in the APP-PS1 mice (Fig. 2D, F). Therefore, differences in the number of lysosomes per astrocyte could result from more cells possessing at least one of these organelles, rather than an increased number of lysosomes per astrocytic cell. In addition, we observed an increased number of lipid bodies (Control 1.429 ± 4.161 lipids per astrocyte *vs* Far AD 4.161 ± 0.7706 lipids per astrocyte, *p* = 0.0009), and percentage of astrocytes containing at least one lipid body (Control 57.14 ± 9.524% of astrocytes *vs* Far AD 83.87 ± 6.715% of astrocytes, *p* = 0.0424) in the APP-PS1 mice compared to C57BL/6J control mice (Fig. 2G–H). Overall, these findings indicate that astrocytes in the *stratum radiatum* of 20-month-old APP-PS1 male mice exhibit more mature lysosomes, accumulated lipid bodies, and increased interactions with synaptic elements compared to age-matched C57BL/6J mice (see Tables 1 and 2).

**Table 1:** Absolute ultrastructural analysis of typical astrocytes far from Aß plaques/dystrophic neurites and in aged APP-PS1 mice compared to age-matched C57BL/6 mice in the *stratum radiatum* of the ventral hippocampus CA1

	Control Mean ± SEM (Min–Max)	AD Mean ± SEM (Min–Max)
Primary lysosomes (*n*)	1.179 ± 0.2523 (0.000–5.000)	0.6129 ± 0.1950 (0.000–4.000)
Secondary lysosomes (*n*)	1.607 ± 0.4435 (0.000–10.00)	1.032 ± 0.2475 (0.000–5.000)
Tertiary lysosomes (*n*)	1.464 ± 0.3313 (0.000–6.000)	2.742 ± 0.3934 * (0.000–8.000)
All lysosomes (*n*)	4.179 ± 0.6384 (0.000–12.00)	4.032 ± 0.5893 (0.000–12.00)
Lipid bodies (*n*)	1.429 ± 0.4224 (0.000–11.00)	4.161 ± 0.7706 *** (0.000–19.00)
Altered mitochondria (*n*)	2.356 ± 0.2780 (0.000–6.000)	2.387 ± 0.2847 (0.000–6.000)
Elongated mitochondria (*n*)	3.286 ± 0.4903 (0.000–10.00)	3.419 ± 0.5283 (0.000–11.00)
Partially digested phagosomes (*n*)	2.857 ± 0.5213 (0.000–12.00)	3.710 ± 0.5230 (0.000–11.00)
Fully digested phagosomes (*n*)	2.929 ± 0.4482 (0.000–7.000)	4.258 ± 0.7139 (0.000–14.00)
All phagosomes (*n*)	5.786 ± 0.7226 (0.000–15.00)	7.968 ± 1.160 (0.000–24.00)
Association with myelinated axons (*n*)	1.464 ± 0.4755 (0.000–9.000)	1.065 ± 0.3437 (0.000–9.000)
Axon terminals (*n*)	16.25 ± 1.729 (4.000–42.00)	22.29 ± 2.089 * (6.000–43.00)
Dendritic spines (*n*)	1.429 ± 0.2020 (0.000–4.000)	2.194 ± 0.2384 * (0.000–4.000)
All synaptic contacts (*n*)	20.68 ± 2.244 (0.000–54.00)	29.06 ± 2.587 * (8.000–54.00)
Dilated ER (*n*)	4.143 ± 0.5767 (1.000–13.00)	5.129 ± 0.6255 (0.000–16.00)
Autophagosomes (*n*)	0.5000 ± 0.1585 (0.000–3.000)	0.7742 ± 0.1518 (0.000–3.000)
Cell area (µm^2^)	57.65 ± 3.380 (35.58–107.1)	64.01 ± 5.421 (26.18–160.1)
Cytoplasm area (µm^2^)	31.31 ± 3.143 (12.81–84.33)	38.54 ± 4.795 (9.553–142.5)
Nucleus area (µm^2^)	26.34 ± 1.598 (10.05–41.75)	25.47 ± 1.973 (8.942–53.39)
Cell perimeter (µm)	62.65 ± 3.603 (33.26–111.6)	66.83 ± 3.841 (27.89–104.0)
Nucleus perimeter (µm)	21.80 ± 1.036 (12.30–36.71)	20.65 ± 0.9511 (12.01–28.53)
Circularity (a.u.)	0.2114 ± 0.01728 (0.08100–0.4420)	0.1977 ± 0.01601 (0.08200–0.4680)
AR (a.u.)	2.178 ± 0.1097 (1.163–3.610)	2.467 ± 0.1657 (1.206–4.273)
Solidity (a.u.)	0.6219 ± 0.02208 (0.4170–0.8290)	0.6157 ± 0.02169 (0.3600–0.8690)

*n* number, *a.u.* arbitrary unit, *ER* endoplasmic reticulum, and *p*-values of statistically significant tests are highlighted with an asterisk symbol

Data reported are shown as number per cell and expressed as means ± SEM in addition to the minimum and maximum value obtained

*p < 0.05, ***p < 0.001 using a non-parametric Mann–Whitney test. Statistical tests were performed on *n* = 8–11 astrocytes per animal in *N* = 3 mice/group, for a total of 59 cell bodies analyzed

**Table 2:** Relative ultrastructural analysis of typical astrocytes far from Aß plaques/dystrophic neurites in aged APP-PS1 mice compared to age-matched C57BL/6 mice in the *stratum radiatum* of the ventral hippocampus CA1

	Control Mean ± SEM	Far AD Mean ± SEM
Primary lysosomes (%)	57.14 ± 9.524	32.36 ± 8.535
Secondary lysosomes (%)	46.43 ± 9.598	51.61 ± 9.124
Tertiary lysosomes (%)	53.57 ± 9.598	77.42 ± 7.634
Lipid bodies (%)	57.14 ± 9.524	83.87 ± 6.715*
Altered mitochondria (%)	85.71 ± 6.734	90.32 ± 5.398
Elongated mitochondria (%)	96.43 ± 3.571	90.32 ± 5.398
Glycogen granules (%)	75.00 ± 8.333	87.10 ± 6.121
Dilated ER (%)	100.0 ± 0.000	96.77 ± 3.226
Nuclear indentation (%)	10.71 ± 5.952	16.13 ± 6.715

% percent, *a.u.* arbitrary unit, *ER* endoplasmic reticulum

Data reported are shown as % of cells positive for at least one of the elements analyzed for each category and expressed as means ± SEM. The statistical test performed was a non-parametric Mann–Whitney test with *p < 0.05 . Statistical tests were performed on *n* = 8–11 astrocytes per animal in *N* = 3 mice/group, for a total of 59 cell bodies analyzed

### Typical astrocytes in the hippocampal CA1 *stratum lacunosum-moleculare *of aged APP-PS1 *vs* age-matched C57BL/6J mice present increased synaptic contacts and phagolysosomal activity

**Fig. 3:**
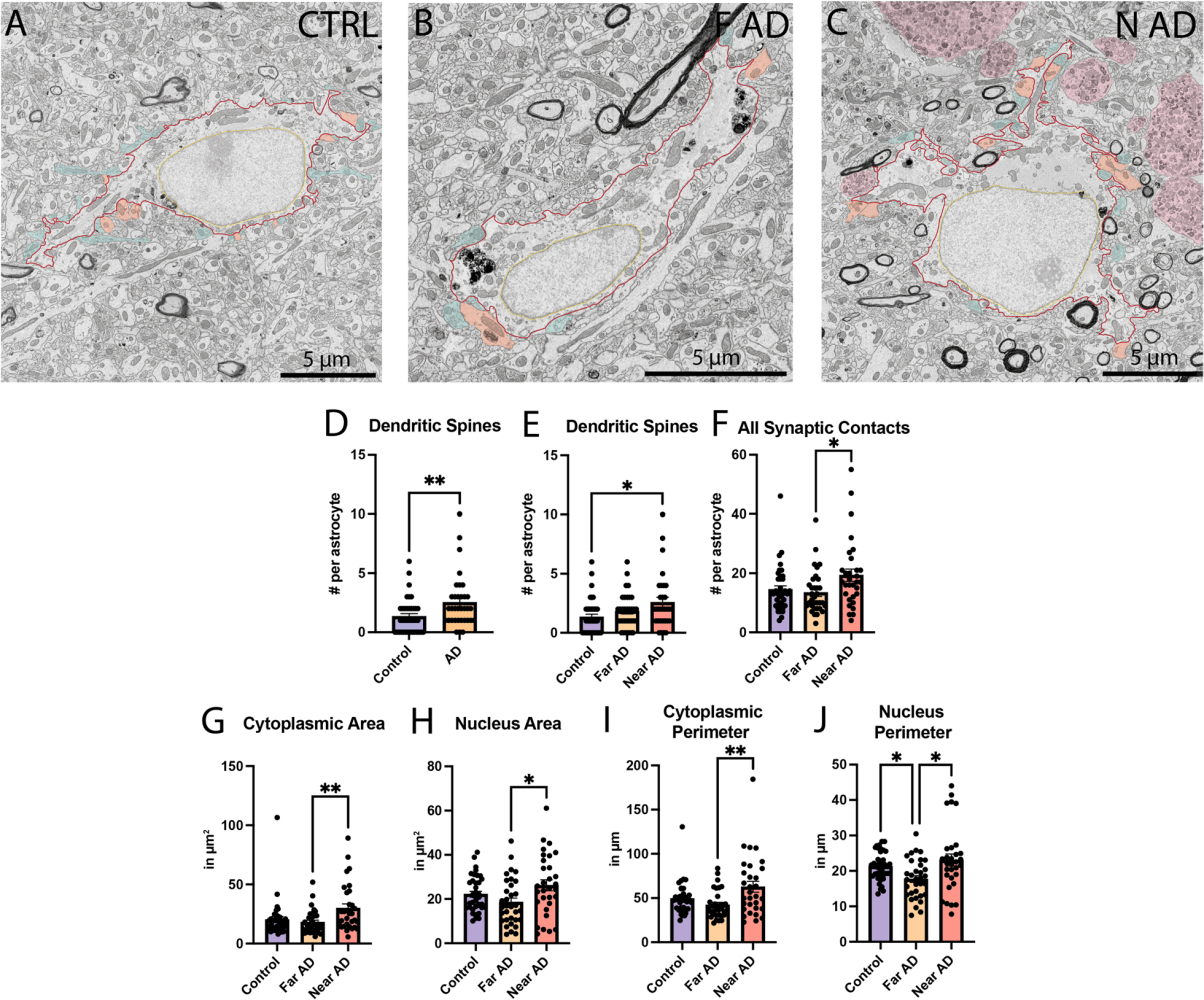
Parenchymal interactions of typical astrocytes and shape descriptors in the *stratum lacunosum-moleculare.* Representative 5 nm per pixel of resolution scanning electron microscopy images acquired in the ventral hippocampus CA1 *stratum lacunosum-moleculare* of 20-month-old C57BL/6J male mice (**A**) and APP-PS1 male mice far (**B**) and near (**C**) Aß plaques/dystrophic neurites. Quantitative graphs represent the number of direct contacts with dendritic spines (**D**) per genotype (APP-PS1 *vs* C57BL/6J), and contacts with dendritic spines (**E**) and with synaptic elements (**F**) when spatially separating astrocytes between locations near *vs* far Aß plaques/dystrophic neurites. Quantitative graphs represent the shape descriptors of the astrocytic cell bodies, including **G** cytoplasmic area, **H** nucleus area, **I** cytoplasmic perimeter, and **J** nucleus perimeter. Data are shown as individual dots and are expressed as means ± S.E.M. *p < 0.05, **p < 0.01, using a non-parametric Mann–Whitney test for the comparison of dendritic spines in **D**, and a Kruskal–Wallis test with a Dunn’s post hoc for all other graphs shown. Statistical tests were performed on *n* = 8–12 astrocytes per animal in *N* = 3 mice/group, for a total of 102 cell bodies analyzed. red outline = plasma membrane, yellow outline = nuclear membrane, blue pseudo-coloring = axon terminals, orange pseudo-coloring = dendritic spines

We next pursued our ultrastructural investigation of typical astrocytes in the *stratum lacunosum-moleculare* (Fig. 3A–C). As in the *stratum radiatum*, we observed an increased prevalence of direct contacts between astrocytes and dendritic spines in the APP-PS1 mice compared to C57BL/6J control mice (Control 1.342 ± 0.2394 contacts per astrocyte *vs* AD 2.529 ± 0.3829 contacts per astrocyte,* p* = 0.0055) (Fig. 3D). When discriminating further the astrocytes based on their proximity to Aß plaques/dystrophic neurites [far (Far AD) *vs* near (Near AD) Aß plaques/dystrophic neurites], astrocytes near Aß plaques/dystrophic neurites were found to be mainly responsible for these increased contacts with dendritic spines (Control 1.342 ± 0.2394 contacts per astrocyte *vs* Near AD 2.5821 ± 0.4219 contacts per astrocyte, *p* = 0.0240) (Fig. 3E). In addition, we observed an overall reduction in the direct contacts with synapses for astrocytes located far *versus* near Aß plaques/dystrophic neurites (Far AD 13.45 ± 1.261 contacts per astrocyte *vs* Near AD 19.35 ± 2.071 contacts per astrocyte, *p* = 0.0340) (Fig. 3E).

However, unlike the astrocytes analyzed in the *stratum radiatum*, we measured in the current layer an increase in the area and perimeter of both the cytoplasm and nucleus for astrocytes located near *versus* far from Aß plaques/dystrophic neurites, which could at least partly explain their increased prevalence of synaptic interactions (cytoplasmic area without nucleus—Far AD 18.02 ± 1.731 µm^2^ *vs* Near AD 29.91 ± 3.601 µm^2^, *p* = 0.0083; nuclear area—Far AD 18.64 ± 1.822 µm^2^ *vs* Near AD 26.23 ± 2.486 µm^2^, *p* = 0.0289; cytoplasmic perimeter—Far AD 42.30 ± 2.750 µm *vs* Near AD 62.73 ± 6.112 µm, *p* = 0.0057; nucleus perimeter—Far AD 17.72 ± 0.9569 µm *vs* Near AD 23.07 ± 1.685 µm, *p* = 0.0135) (Fig. 3G–J). These differences are in line with the findings from previous studies that highlight an atrophy of astrocytes observed far from Aß plaques compared to their hypertrophy near Aß plaques in mouse models of AD pathology [104–106], a morphological shift suggested to be associated with the appearance of Aß plaques within their microenvironment [104].

**Fig. 4:**
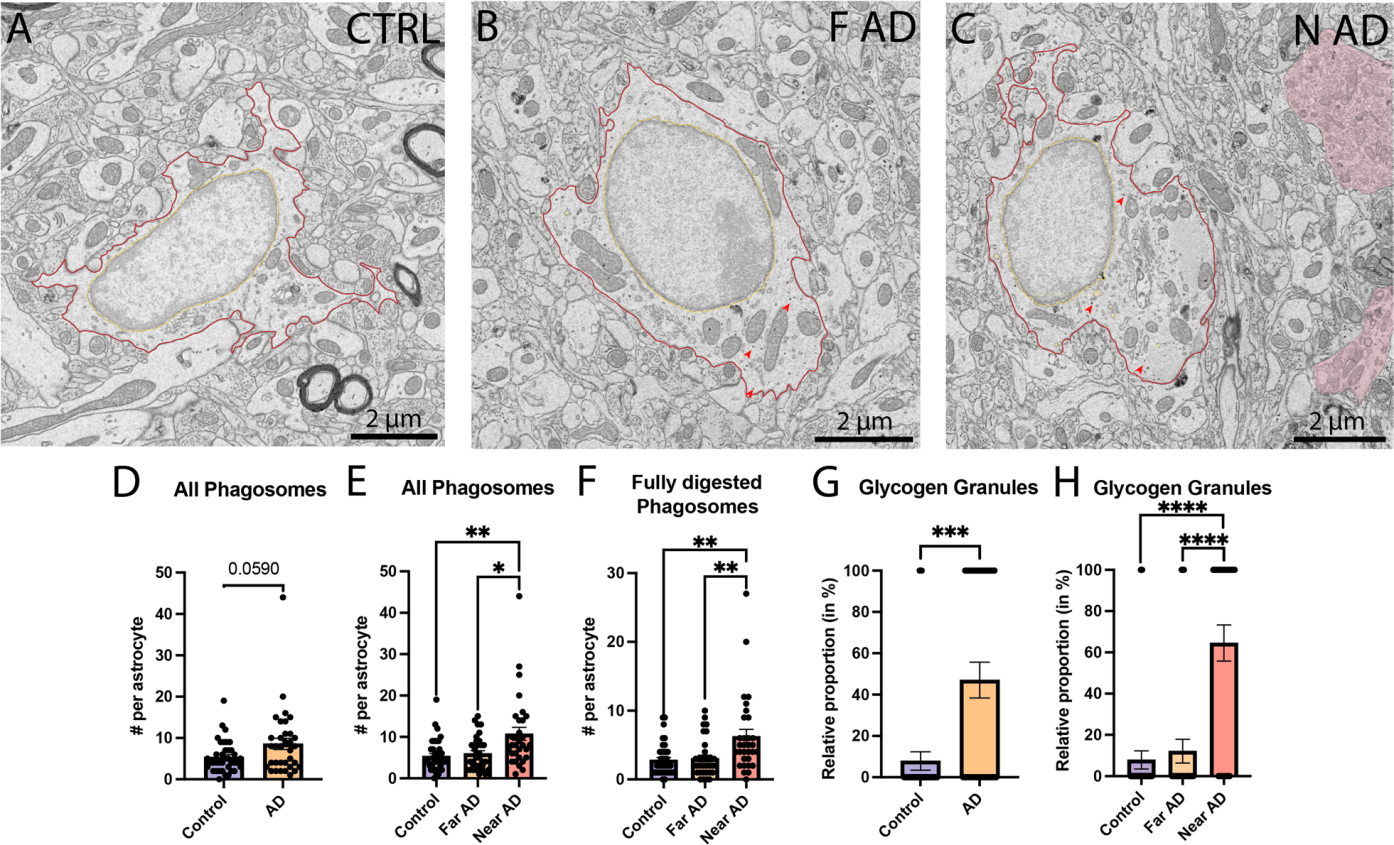
Intracellular contents of typical astrocytes in the *stratum lacunosum-moleculare.* Representative 5 nm per pixel of resolution scanning electron microscopy images acquired in the ventral hippocampus CA1 *stratum lacunosum-moleculare* of 20-month-old C57BL/6J male mice (**A**) and APP-PS1 male mice far (**B**) and near (**C**) Aß plaques/dystrophic neurites. Quantitative graphs representing the number of phagosomes **D** per genotype (APP-PS1 *vs* C57BL/6J) and **E** per proximity to Aß plaques/dystrophic neurites. The number of fully digested phagosomes per astrocytic cell body based on the proximity to Aß plaques/dystrophic neurites is shown in **F**. Quantitative graphs represent the number of cells positive for glycogen granules per genotype (**G**) and per proximity to Aß plaques/dystrophic neurites (**H**). Data are shown as individual dots and are expressed as mean ± S.E.M. *p < 0.05, **p < 0.01, ***p < 0.001, ****p <  0.0001 using a non-parametric Mann–Whitney test for the comparison of phagosomes (**D**) and glycogen granules (**H**), and a Kruskal–Wallis test with a Dunn’s post hoc for all other graphs shown. Statistical tests were performed on *n* = 8–12 astrocytes per animal in *N* = 3 mice/group, for a total of 102 cell bodies analyzed. Red outline = plasma membrane, yellow outline = nuclear membrane, red arrow = glycogen granules, yellow pseudo-coloring = fully digested phagosomes

In terms of intracellular contents, our analysis of typical astrocytes located in the *stratum lacunosum-moleculare* (Fig. 4A–C) further revealed a tendency for an increase in all phagosomes (fully and partially digested phagosomes) in the APP-PS1 mice compared to C57BL/6J control mice (Control 5.368 ± 0.6191 phagosomes per astrocyte *vs* AD 8.5588 ± 1.364 phagosomes per astrocyte, *p* = 0.0590) (Fig. 4D). When we investigated the driving force behind this tendency (i.e., near *vs* far from Aß plaques/dystrophic neurites), we found a significant increase in all phagosomes (fully and partially digested phagosomes) only in astrocytes near Aß plaques/dystrophic neurites, compared to both astrocytes far from Aß plaques/dystrophic neurites in APP-PS1 mice and astrocytes in C57BL/6J control mice (Control 5.368 ± 0.6191 phagosomes per astrocyte *vs* Near AD 10.74 ± 1.573 phagosomes per astrocyte, *p* = 0.0019; Far AD 5.970 ± 0.7010 phagosomes per astrocyte *vs* Near AD 10.74 ± 1.573 phagosomes per astrocyte, *p* = 0.0160) (Fig. 4E). This increased number of phagosomes per astrocyte located near Aß plaques/dystrophic neurites was identified specifically for the fully digested phagosomes (Control 2.842 ± 0.3781 phagosomes per astrocyte *vs* Near AD 6.258 ± 1.017 phagosomes per astrocyte, *p* = 0.0016; Far AD 3.061 ± 0.4766 phagosomes per astrocyte *vs* Near AD 6.258 ± 1.017 phagosomes per astrocyte, *p* = 0.0046) (Fig. 4F). In short, both the *strata lacunosum-moleculare* and *radiatum* showed an increased activity of the phagolysosomal pathway in aged 20-month-old APP-PS1 male mice compared to age-matched C57BL/6J controls, resulting in an increased prevalence of mature lysosomes and fully digested phagosomes, respectively.

In our analysis, we also examined glycogen granules, a carbohydrate storage that can be broken down to glucose through glycolysis, and which was shown to be crucial in astrocytes for learning and memory [107, 108], and associated with aging in human brain samples [109]. Glycogen granules were shown to be located within astrocytic processes, notably those in proximity to dendritic spines and axon terminals in the hippocampus and sensorimotor cortex of rodents [110–112]. In the current study, while there were no differences detected in the *stratum radiatum*, more astrocytes in the *stratum lacunosum-moleculare* were found to contain glycogen granules in the APP-PS1 mice compared to C57BL/6J control mice (Control 7.895 ± 4.433% of astrocytes *vs* AD 47.06 ± 8.689% of astrocytes, *p* = 0.0002). Indeed, close to half of all astrocytes in APP-PS1 mice were positive for glycogen granules compared to nearly 8% in C57BL/6J control mice (Fig. 4G). When astrocytes were spatially separated between locations near *vs* far Aß plaques/dystrophic neurites, the majority of astrocytes with glycogen granules were found near *versus* far from Aß plaques/dystrophic neurites (Control 7.895 ± 4.433% of astrocytes *vs* Near AD 64.52 ± 8.736% of astrocytes, *p* < 0.0001; Far AD 12.12 ± 5.770% of astrocytes *vs* Near AD 64.52 ± 8.736% of astrocytes, *p* < 0.0001) (see Tables 3 and 4).

**Table 3:** Absolute ultrastructural analysis of typical astrocytes near *vs* far from Aß plaques/dystrophic neurites in aged APP-PS1 mice compared to age-matched C57BL/6 mice in the *stratum lacunosum-moleculare* of the ventral hippocampus CA1

	C57BL/6J	APP-PS1	
	Control Mean ± SEM (Min–Max)	Far AD Mean ± SEM (Min–Max)	Near AD Mean ± SEM (Min–Max)
Primary lysosomes (*n*)	0.6316 ± 0.1432 (0.000–3.000)	0.6061 ± 0.1737 (0.000–4.000)	0.5484 ± 0.1379 (0.000–3.000)
Secondary lysosomes (*n*)	1.211 ± 0.2776 (0.000–7.000)	0.6364 ± 0.1837 (0.000–4.000)	0.8065 ± 0.2384 (0.000–6.000)
Tertiary lysosomes (*n*)	0.4737 ± 0.1398 (0.000–4.000)	0.6061 ± 0.1737 (0.000–3.000)	0.6774 ± 0.2194 (0.000–4.000)
All lysosomes (*n*)	2.316 ± 0.3580 (0.000–9.000)	1.848 ± 0.3202 (0.000–6.000)	2.032 ± 0.3724 (0.000–9.000)
Lipid bodies (*n*)	2.184 ± 0.4663 (0.000–11.00)	2.909 ± 0.7049 (0.000–14.00)	3.839 ± 0.9860 (0.000–22.00)
Altered mitochondria (*n*)	0.8947 ± 0.1545 (0.000–3.000)	0.6061 ± 0.1625 (0.000–4.000)	1.065 ± 0.2172 (0.000–4.000)
Elongated mitochondria (*n*)	2.579 ± 0.4869 (0.000–17.00)	1.939 ± 0.2818 (0.000–6.000)	3.129 ± 0.6077 (0.000–13.00)
All mitochondria (*n*) *	13.39 ± 1.095 (1.000–40.00)	12.79 ± 1.004 (2.000–26.00)	19.55 ± 2.270 (1.000–57.00)
Partially digested phagosomes (*n*)	2.526 ± 0.3533 (0.000–10.00)	2.909 ± 0.4091 (0.000 ± 8.000)	4.484 ± 0.7212 (0.000–17.000)
Fully digested phagosomes (*n*)***	2.842 ± 0.3781 (0.000–9.000)	3.061 ± 0.4766 (0.000–10.00)	6.258 ± 1.017 &&!! (0.000–27.00)
All phagosomes (*n*)**	5.368 ± 0.6191 (0.000–19.00)	5.970 ± 0.7010 (1.000–15.00)	10.74 ± 1.573 &&! (1.000–44.00)
Association with myelinated axons (*n*)	1.763 ± 0.4953 (0.000–14.00)	1.576 ± 0.4013 (0.000–9.000)	1.258 ± 0.4121 (0.000–10.00)
Axon terminals (*n*)	9.579 ± 0.8614 (2.000–31.00)	8.727 ± 1.026 (1.000–31.00)	12.26 ± 1.392 (2.000–33.00)
Dendritic spines (*n*) *	1.342 ± 0.2394 (0.000–6.000)	1.848 ± 0.2579 (0.000–6.000)	2.581 ± 0.4219 & (0.000–10.00)
All synaptic contacts (*n*)*	14.50 ± 1.245 (4.000–46.00)	13.45 ± 1.261 (3.000–38.00)	19.35 ± 2.071! (4.000–51.00)
Dilated ER (*n*)	2.368 ± 0.4967 (0.000–16.00)	1.303 ± 0.2769 (0.000–7.000)	3.129 ± 0.9120 (0.000–20.00)
Non-dilated ER (*n*)	16.82 ± 1.976 (2.000–73.00)	12.30 ± 1.253 (4.000–29.00)	18.06 ± 2.313 (2.000–48.00)
Dilated Golgi apparatus (*n*)*	1.000 ± 0.2444 (0.000–8.000)	0.2727 ± 0.007873 (0.000–1.000)	0.7097 ± 0.1552 (0.000–3.000)
Non-dilated Golgi apparatus (*n*)	2.184 ± 0.4265 (0.000–13.00)	1.636 ± 0.2254 # (0.000–4.000)	2.968 ± 0.4436 (0.000–9.000)
Autophagosomes (*n*)	0.2105 ± 0.09359 (0.000–3.000)	0.5758 ± 0.1742 (0.000–4.000)	0.2903 ± 0.09497 (0.000–2.000)
Cell area (µm^**2**^)**	42.53 ± 2.774 (20.19–120.0)	36.66 ± 2.803 (14.73 ± 73.60)	56.15 ± 4.641!!! (16.77–130.2)
Cytoplasmic area (µm^**2**^) **	20.32 ± 2.584 (8.028–106.5)	18.02 ± 1.731 (5.983–51.94)	29.91 ± 3.601 !! (5.633–89.16)
Nucleus area (µm^**2**^)*	22.21 ± 1.290 (10.12–41.16)	18.64 ± 1.822 (4.045–46.22)	26.23 ± 2.486! (4.264–61.13)
Cell perimeter (µm)**	49.50 ± 2.905 (24.79–130.6)	42.30 ± 2.750 (21.78–83.58)	62.73 ± 6.112!! (22.71–184.5)
Nucleus perimeter (µm)**	21.35 ± 0.6547 (13.53–28.32)	17.72 ± 0.9569 (7.461–30.47)	23.07 ± 1.685 (7.796–44.02)
AR (a.u.)	2.270 ± 0.1555 (1.051–5.068)	2.217 ± 0.1752 # (1.087–5.509)	2.295 ± 0.1771! (1.204–5.750)
Circularity (a.u.)	0.2468 ± 0.01565 (0.08800–0.4980)	0.2940 ± 0.02280 (0.1090–0.6330)	0.2483–0.02722 (0.04800–0.5960)
Solidity (a.u.)	0.6741 ± 0.02028 (0.3550–0.8990)	0.7146 ± 0.02278 (0.4140–0.8980)	0.6930 ± 0.02512 (0.4070–0.9560)

*n* number, *a.u.* arbitrary unit, *ER* endoplasmic reticulum, and *p*-values of statistically significant tests are highlighted with various symbols (!, #, &)

Data reported are shown as number per cell and expressed as means ± SEM in addition to the minimum and maximum value obtained

*p < 0.05, **p < 0.01, ***p < 0.001 using a Kruskal–Wallis test with a Dunn’s multiple comparisons post hoc test. * *p* value summary, ! Near *vs* Far AD, & Far AD *vs* C57BL/6J, # Far AD *vs* C57BL/6J. Statistical tests were performed on *n* = 8–12 astrocytes per animal in *N* = 3 mice/group, for a total of 102 cell bodies analyzed

**Tabl﻿e 4:** Relative ultrastructural analysis of typical astrocytes near *vs* far from Aß plaques/dystrophic neurites in aged APP-PS1 mice compared to age-matched C57BL/6 mice in the *stratum lacunosum-moleculare* of the ventral hippocampus CA1

	C57BL/6J	APP-PS1	
	Control Mean ± SEM	Far AD Mean ± SEM	Near AD Mean ± SEM
Primary lysosomes (%)	39.47 ± 8.036	33.33 ± 8.333	41.94 ± 9.009
Secondary lysosomes (%)	50.00 ± 8.220	36.36 ± 8.504	45.16 ± 9.086
Tertiary lysosomes (%)	31.58 ± 7.642	30.30 ± 8.124	29.03 ± 8.287
Lipid bodies (%)	55.26 ± 8.174	51.52 ± 8.835	61.29 ± 8.893
Altered mitochondria (%)	55.26 ± 8.174	42.42 ± 8.737	61.29 ± 8.893
Elongated mitochondria (%)	84.21 ± 5.995	81.82 ± 6.818	77.42 ± 7.634
Glycogen granules (%) ****	7.895 ± 4.433	12.12 ± 5.770	64.52 ± 8.736 &&&& !!!!
Dilated ER (%)	71.05 ± 7.456	63.64 ± 8.504	80.65 ± 7.213
Nuclear indentation (%)	13.16 ± 5.557	3.030 ± 3.030	19.35 ± 7.213

% percent, a.u. arbitrary unit, ER endoplasmic reticulum, *p*-values of statistically significant tests are highlighted with various symbols (!, &) with * indicating *p *value summary

Data reported are shown as % of cells positive for at least one of the elements analyzed for each category and expressed as means ± SEM

****p < 0.0001 using a Kruskal–Wallis test with Dunn’s multiple comparisons post hoc test. *p value summary, ! Near *vs* Far AD, & Far AD *vs* C57BL/6J. Statistical tests were performed on *n* = 8–12 astrocytes per animal in *N* = 3 mice/group, for a total of 102 cell bodies analyzed

### Dark astrocytes in the hippocampal CA1* stratum lacunosum-moleculare* of aged APP-PS1 *vs* age-matched C57BL/6J mice present similar densities and interactions with the vasculature

**Fig. 5:**
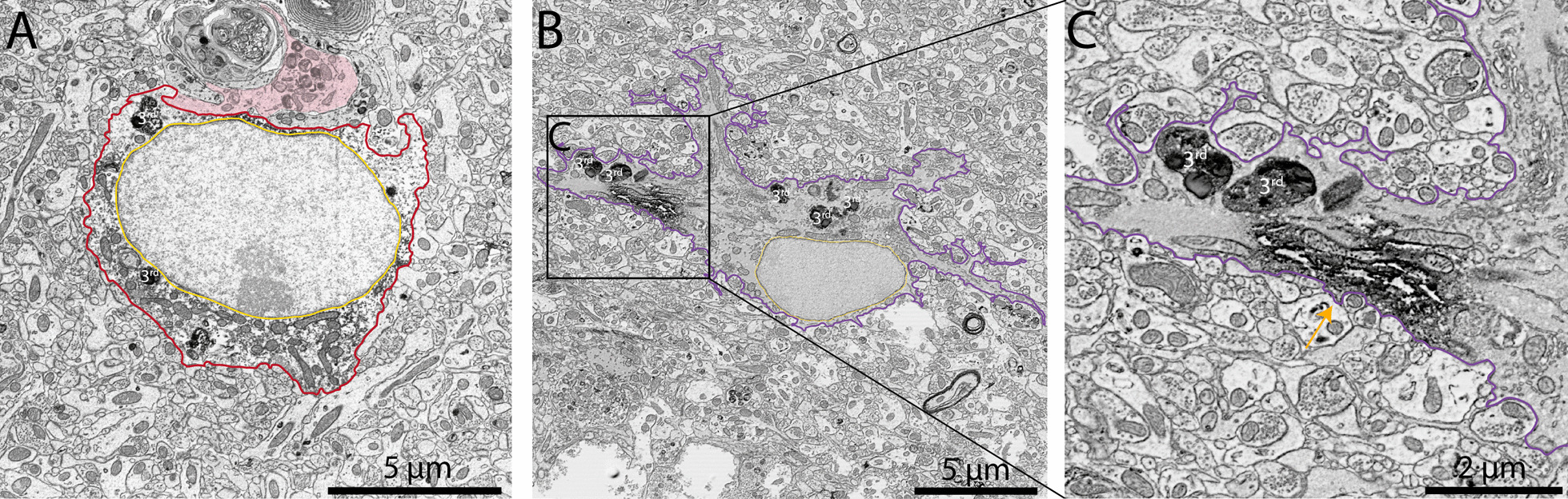
Immunostaining for GFAP in typical and dark astrocytes of the *stratum lacunosum-moleculare.* Representative 5 nm per pixel and 1 nm per pixel of resolution scanning electron microscopy images showing a typical (**A**) and dark astrocyte (**B**, **C**) immunostained with glial fibrillary acidic protein (GFAP) in the ventral hippocampus CA1 *stratum lacunosum-moleculare* of 20-month-old APP-PS1 male mice. In **A** a typical astrocyte, denoted by its electron-lucent cyto- and nucleoplasm, is immunopositive for GFAP. In **B** an electron-dense dark astrocyte with hyper-ramifications and several tertiary lysosomes is immunostained for GFAP. In **C** a close-up of the dark astrocyte where the GFAP staining is indicated with an orange arrow. Yellow outline = nuclear membrane, purple outline = dark astrocytic cytoplasm, red outline = typical astrocytic cytoplasm, orange arrow = GFAP immunostaining in dark astrocyte, pink pseudo-coloring = dystrophic neurites, purple pseudo-coloring = amyloid beta plaques, 3rd = tertiary lysosomes

**Fig. 6:**
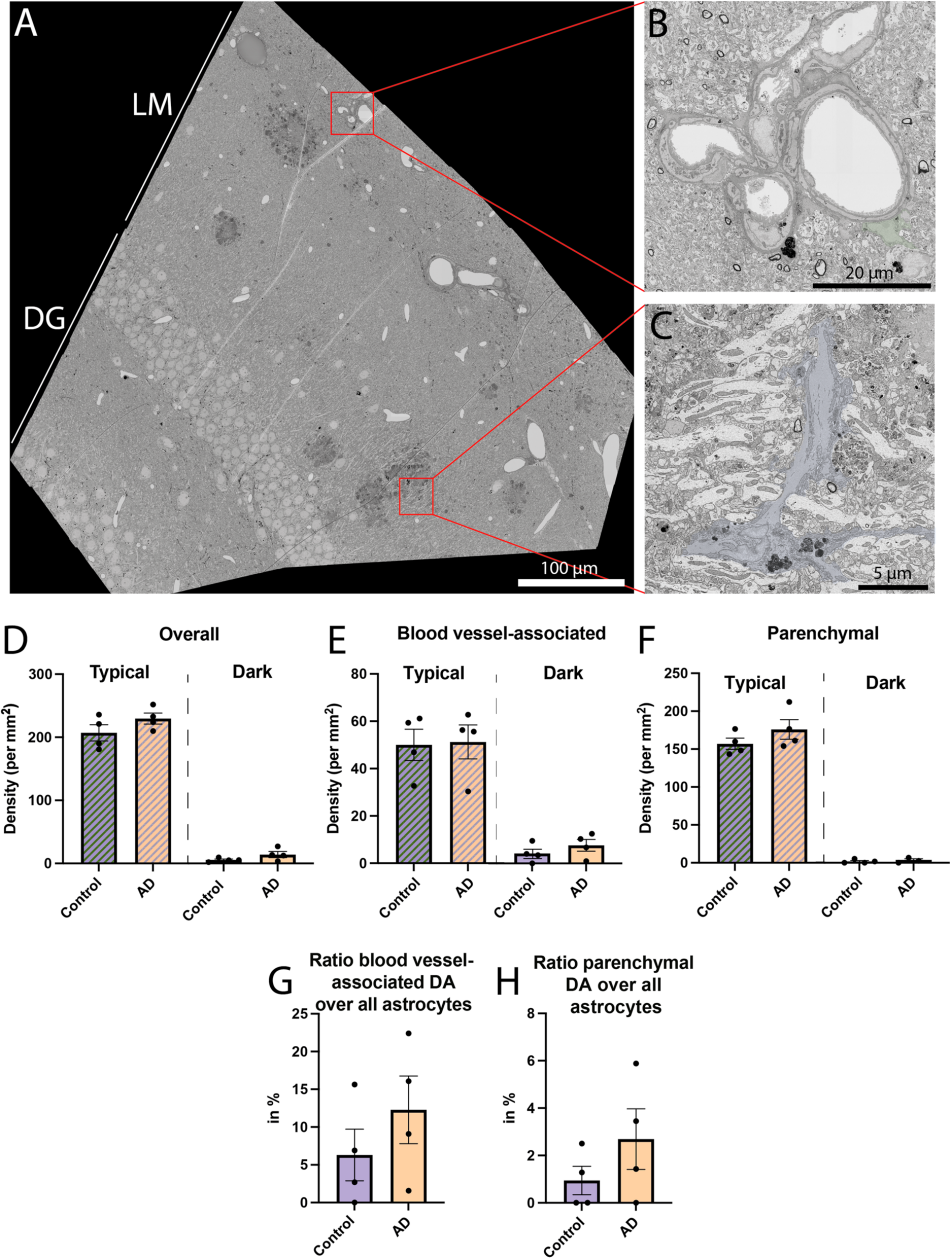
Density of dark and typical astrocytes in the *stratum lacunosum-moleculare*. Representative 25 nm per pixel (**A**) and 5 nm per pixel (**B**, **C**) of resolution scanning electron microscopy images of dark astrocytes associated with blood vessels (**B**) and with the parenchyma (**C**) from a 20-month-old APP-PS1 male mouse. Quantitative graphs represent the astrocytic density defined ultrastructurally (e.g., via their intermediate filaments, angular processes) and their electron-dense ultrastructure (dark) or electron-lucent (typical) appearance (**D**) in 20-month-old C57BL/6J *vs* APP-PS1 male mice. Typical and dark astrocytes in these mice were further categorized based on their association (**E**) or lack of association (**F**) with blood vessels in the plane of view. The ratio of dark astrocytic cells associated with a blood vessel and overall astrocytes (typical and dark) associated with the vasculature is represented (**G**), while the ratio of all dark astrocytes not associated with blood vessels over all astrocytes not associated with blood vessels (typical and dark) is provided (**H**). Data are shown as individual dots and are expressed as mean ± S.E.M using a Welsh test. Statistical tests were performed on n = 4 mice/group (2–6 levels per animal). Green pseudo-coloring = dark astrocyte associated with blood vessels, purple pseudo-coloring = dark astrocyte not associated with blood vessels

While imaging in aged APP-PS1 and C57BL/6J mice, we identified an electron-dense astrocytic state based on their distinct ultrastructural features and located often near the vasculature. We confirmed that the dark astrocytes in the ventral hippocampus CA1 were also immunopositive for GFAP, a marker generally associated with astrocytes termed ‘reactive’ (Fig. 5A–C). Dark astrocytes were previously observed both in rodents (e.g., rat models of brain injury, kainic and pentylenetetrazole treatments, electroshock; mouse embryonic spinal cord culture) [65–67] and human post-mortem brain samples (e.g., brain tumors, brain injury) [61–64]. These cells were described as having hypertrophic electron-dense cell bodies and processes often containing altered mitochondria and glycogen granules [61, 62, 65]. While their roles have remained largely elusive, we further confirmed the presence of a similar electron-dense astrocytic state in the ventral hippocampus CA1 of 20-month-old APP-PS1 and C57BL/6J male mice. We then performed quantitative analysis of their distribution (Fig. 6A–C), and examined whether dark astrocytes interacted more or less often with blood vessels in the *stratum lacunosum-moleculare* of APP-PS1 *vs* C57BL/6J mice, as vascular dysfunction was previously noted in the hippocampus during aging and AD pathology, using human and mouse samples [113–117].

Dark astrocytes were not found exclusively in aged APP-PS1 mice, as they were also observed in age-matched C57BL/6J controls (Control 5.518 ± 1.546 cells per mm^2^ *vs* AD 14.190 ± 4.861 cells per mm^2^, *p* = 0.1721) (Fig. 6D). Typical astrocytes also did not display significant differences in their density between the two genotypes (Control 207.1 ± 12.89 cells per mm^2^ vs AD 229.6 ± 8.802 cells per mm^2^, *p* = 0.2056). In addition, most of the dark astrocytes observed in both conditions were in direct contact with blood vessels (Control 4.265 ± 1.976 cells per mm^2^ *vs* AD 7.666 ± 2.528 cells per mm^2^, *p* = 0.3323) while the density of dark astrocytes not touching a blood vessel in the plane of view was lower (Control 1.757 ± 1.230 cells per mm^2^ *vs* AD 3.795 ± 1.550 cells per mm^2^, *p* = 0.3448) (Fig. 6E–F). Typical astrocytes contacting the basement membrane of a blood vessel were similarly abundant in APP-PS1 mice and C57BL/6J controls (Control 50.02 ± 6.584 cells per mm^2^ *vs* AD 51.26 ± 7.142 cells per mm^2^, *p* = 0.9028), and the same finding was obtained for typical astrocytes that did not contact a blood vessel in the plane of view (Control 157.0 ± 7.386 cells per mm^2^ *vs* AD 176.0 ± 12.92 cells per mm^2^, *p* = 0.2614).

**Fig. 7:**
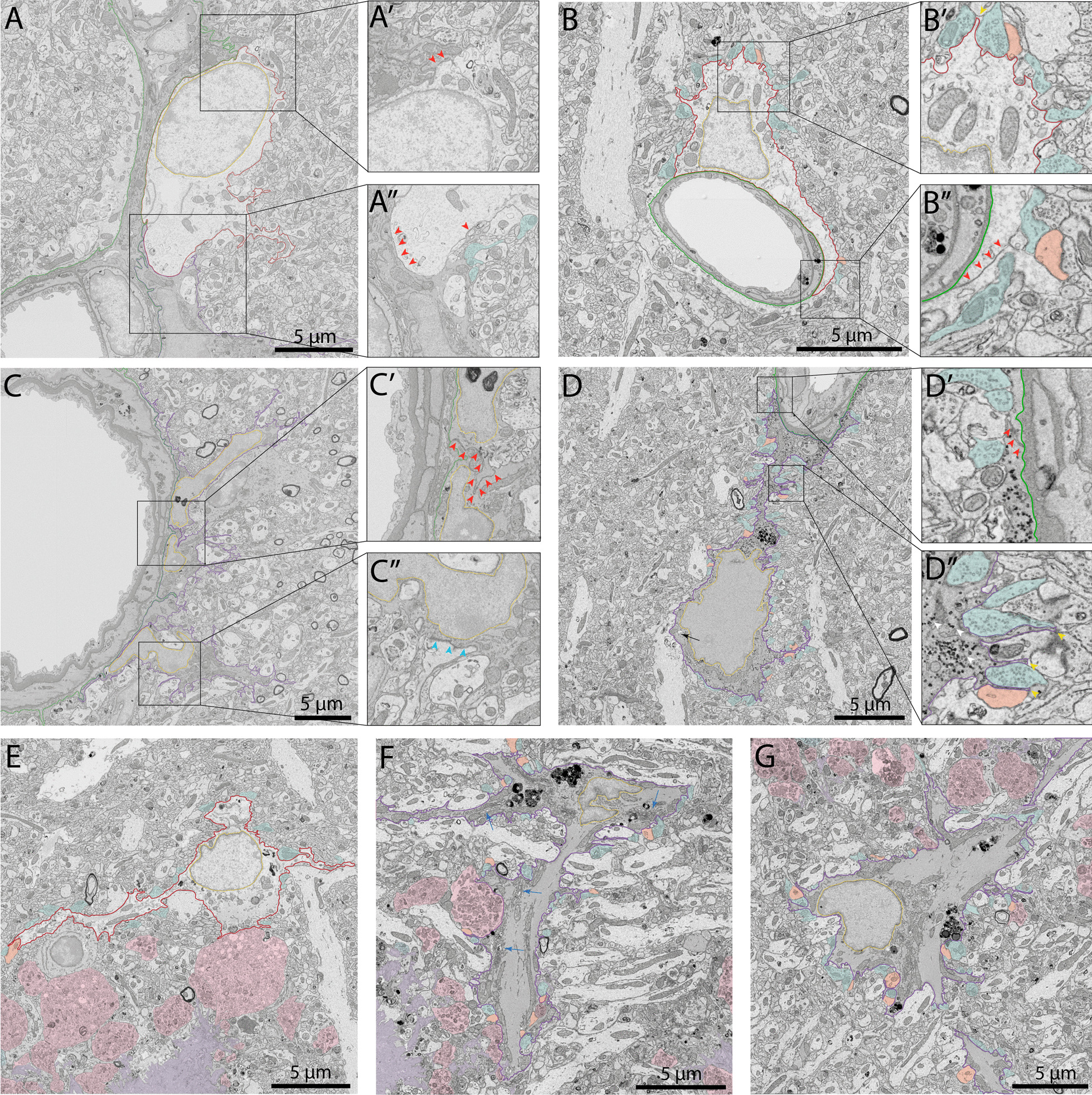
Ultrastructural characterization of dark and typical astrocytes. Representative 5 nm per pixel of resolution scanning electron microscopy images of dark and typical astrocytes acquired in the ventral hippocampus CA1 *stratum lacunosum-moleculare* of 3- to 4-month-old C57BL/6J male mice (**A** and **C**) and *stratum lacunosum-moleculare* of aged APP-PS1 20-month-old male mice (**B**–**G**). In A′, red arrows identify the electron-dense interface between two typical astrocytic elements filled with gap junctions. In A″, black arrow identifies intermediate filaments. In B′, a typical astrocyte makes direct contact with dendritic spines and axon terminals. An angular protuberance is identified with a yellow arrow. In B″, the red arrows identify the electron-dense interface. In C′, the electron-dense interface between two dark astrocytic elements is highlighted with a red arrow. In C″, direct contact of dark astrocytes with a dendritic spine is shown with a blue arrow, comparable to the interaction of synaptic elements and typical astrocytes in B″. In D′, the electron-dense interface between two dark astrocytic end-feet is shown with the red arrows. In D″, glycogen granules identified by white arrows, as well as several contacts with dendritic spines and axon terminals. In **E** and **F**, **G** typical and dark astrocytes, respectively, are located near amyloid beta plaques and dystrophic neurites. Dilated Golgi apparatus cisternae identified by a blue arrow are observed. Several lysosomes identified with an asterisk and internalized dystrophic neurites and dendritic spines are shown. Yellow outline = nuclear membrane, purple outline = dark astrocytic cytoplasm, red outline = typical astrocytic cytoplasm, green outline = basement membrane, red arrow = interface between two astrocytic elements, black arrow = intermediate filaments, white arrow = glycogen granules, blue arrow = dilated Golgi apparatus, yellow arrow = angular processes, white asterisk = lysosomes, orange pseudo-coloring = dendritic spines, pink pseudo-coloring = dystrophic neurites, blue pseudo-coloring = axon terminals, purple pseudo-coloring = amyloid beta plaques

When we looked at the ratio of dark astrocytes over all astrocytes in direct contact with a blood vessel, this dark state presented equivalent ratios in APP-PS1 mice and C57BL/6J controls (Control 6.309 ± 3.415% of dark astrocytes *vs* AD 12.28 ± 4.489% of dark astrocytes, *p* = 0.3332). Similar results were obtained for dark astrocytes that were not directly contacting a blood vessel (Control 0.9455 ± 0.5998% of dark astrocytes *vs* AD 2.690 ± 1.278% of dark astrocytes, *p* = 0.2802) (Fig. 6G–H). Overall, there were no significant differences in the density and ratio of dark astrocytes interacting *vs* non-interacting with a blood vessel between aged APP-PS1 and age-matched C57BL/6J mice, indicating that the distribution of these cells at the vasculature and throughout the parenchyma is shared between aging and AD pathology. Moreover, we also observed dark astrocytes in 3- to 4-month-old C57BL/6J mice within the same region, the ventral hippocampus CA1 *stratum lacunosum-moleculare* (Fig. 7D). While the abundance of these dark astrocytes remains to be quantified over time to determine whether they become more abundant during aging, our results suggest that these cells are not exclusive to aging while their appearance is not driven by AD pathology.

### Dark astrocytes in the *strata lacunosum-moleculare* and *radiatum* of 3- to 4-month-old C57BL/6J and 20-month-old APP-PS1 male mice exhibit similar ultrastructural features as typical astrocytes while displaying distinct characteristics

We next performed an ultrastructural characterization of the dark astrocytes among the ventral hippocampus CA1 *strata lacunosum-moleculare* and *radiatum*. We examined the ultrastructural features of dark and typical astrocytes in young (3- to 4-month-old) C57BL/6J male mice and aged (20-month-old) APP-PS1 mice (Fig. 7). This qualitative analysis revealed numerous similarities between the two astrocytic states, which also displayed distinct characteristics, in homeostatic and pathological conditions. Typical astrocytes possessed angular processes [24, 74, 118], often interacting with synaptic elements, and where a dark interface could be observed between two astrocytic end-feet (electron-dense due to being filled with gap junctions) [119–122] (Fig. 7A, B). We observed similar features in the dark astrocytes associated with blood vessels and other parenchymal elements, both for locations near and far from Aß plaques/dystrophic neurites. Indeed, dark astrocytes displayed the same angular processes, which often inserted themselves between pre- and post-synaptic elements of the same synapse (Fig. 7C, D). In addition, we found the same electron-dense interface between the end-feet of two dark perivascular astrocytes, together with a high accumulation of glycogen granules dispersed throughout their cytoplasm (Fig. 7C, D). Dark astrocytes, similar to dark microglia, were further characterized by their electron-dense cytoplasm and nucleoplasm, alongside a partial to total loss of their chromatin pattern, both in 3- to 4-month-old C57BL/6J (Fig. 7C) and 20-month-old APP-PS1 mice (Fig. 7D).

In the parenchyma of aged APP-PS1 mice, dark astrocytes, much like their typical counterparts, were seen interacting extensively with axon terminals, dystrophic neurites, and dendritic spines (Fig. 7E–G). These dark cells were also seen internalizing dystrophic neurites, a feature that was previously observed in typical astrocytes near Aß plaques/dystrophic neurites in 6- and 12-month-old APP-PS1 mice [53]. In addition, several phagosomes, mostly containing axon terminals and dendritic spines, were observed within the dark astrocytic cytoplasm, alongside tertiary lysosomes and lipid bodies (Fig. 7E–G). In our imaging, we captured a single dark astrocyte associated with a blood vessel in the stratum *lacunosum-moleculare* of an aged APP-PS1 mouse at multiple levels, with each image acquired at a distance of 5–6 µm (Additional file 1: Fig. S1). We identified several phagosomes in the dark astrocytic cytoplasm, most of which were dendritic spines and axon terminals, a feature we previously observed in the dark astrocytes found within the parenchyma. Extensive interactions between the dark astrocyte and synaptic elements were observed in the serial images, a feature attributed in part to their thin and angular processes extending among the parenchyma. Like all dark astrocytes imaged, the presence of glycogen granules was observed throughout the images taken of this particular dark astrocytic cell.

**Fig. 8:**
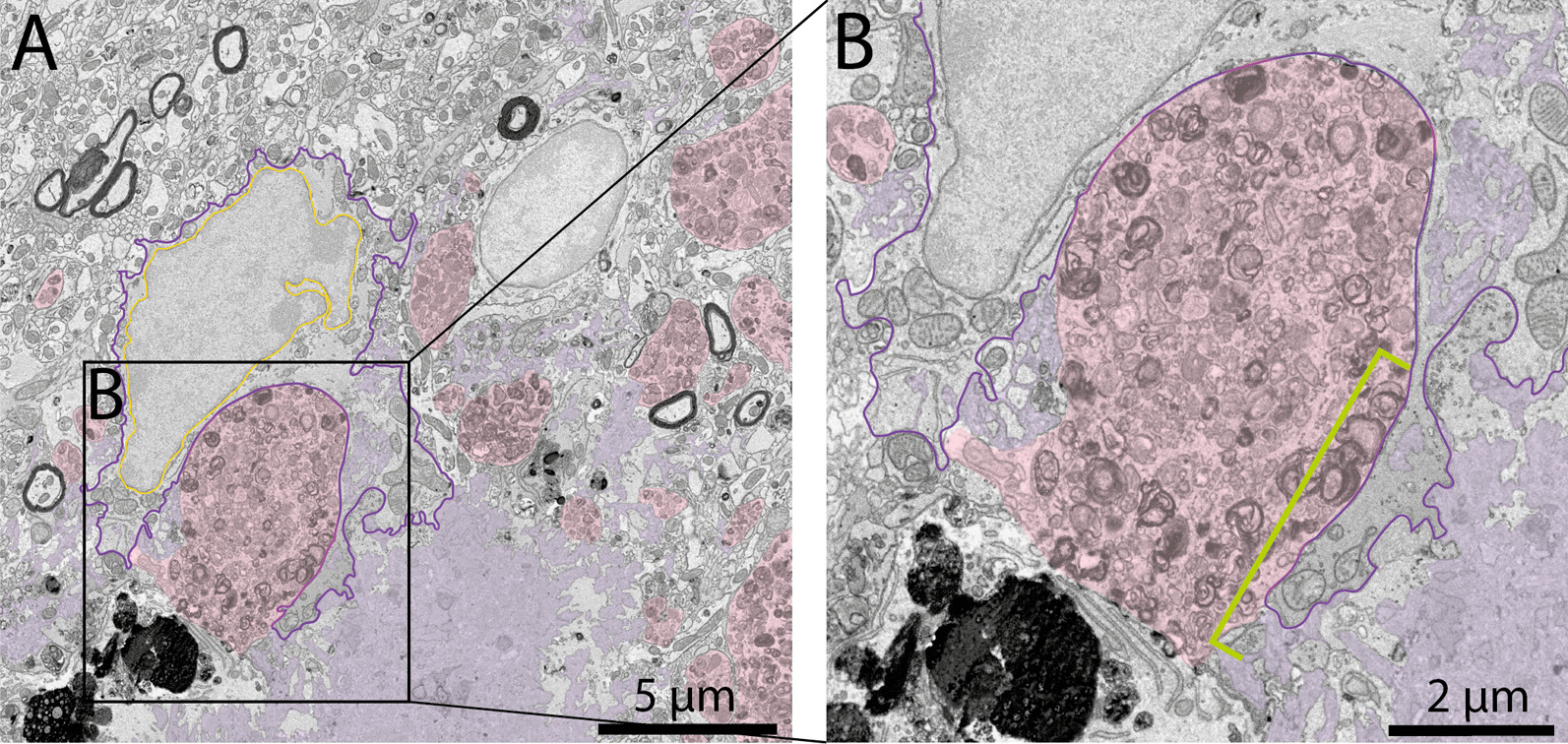
Dark astrocytes are associated with AD hallmarks. Representative 5 nm per pixel of resolution scanning electron microscopy images of a dark astrocyte (**A**, **B**) in the ventral CA1 hippocampus *stratum lacunosum-moleculare* of 20-month-old APP-PS1 male mice. In **A** and **B** the  astrocyte is directly interacting with an Aß plaque where a specific segment (shown in B with a green bar) is becoming electron-dense compared to the rest of the cell. Yellow outline = nuclear membrane, purple outline = dark astrocytic cytoplasm, red outline = typical astrocytic cytoplasm, pink pseudo-coloring = dystrophic neurites, purple pseudo-coloring = amyloid beta plaques, green bar = electron-dense area

Dark astrocytes were further characterized by their markers of cellular stress. Notably, they contained altered mitochondria with swollen cristae that were identified ultrastructurally by an electron-lucent enlargement, mitochondria with a degraded outer membrane, as well as dilated ER and Golgi apparatus cisternae (Fig. 7E–G). The increased electron density within microglial cells was previously hypothesized to be due to the condensation of their cytoplasm related to cellular stress [123]. In addition, Tòth et al. hypothesized that the electron density begins at a specific point in the dark astrocytes where it propagates thereafter throughout the cell [65]. Our observations could support this idea as we found that some astrocytic compartments in direct contact with an Aß plaque and containing fibrillar Aß possessed a more electron-dense appearance (Fig. 8A, B). Therefore, this data could support the view that the electron density of dark astrocytes starts at a specific point which could then spread to the rest of the cell.

**Fig. 9:**
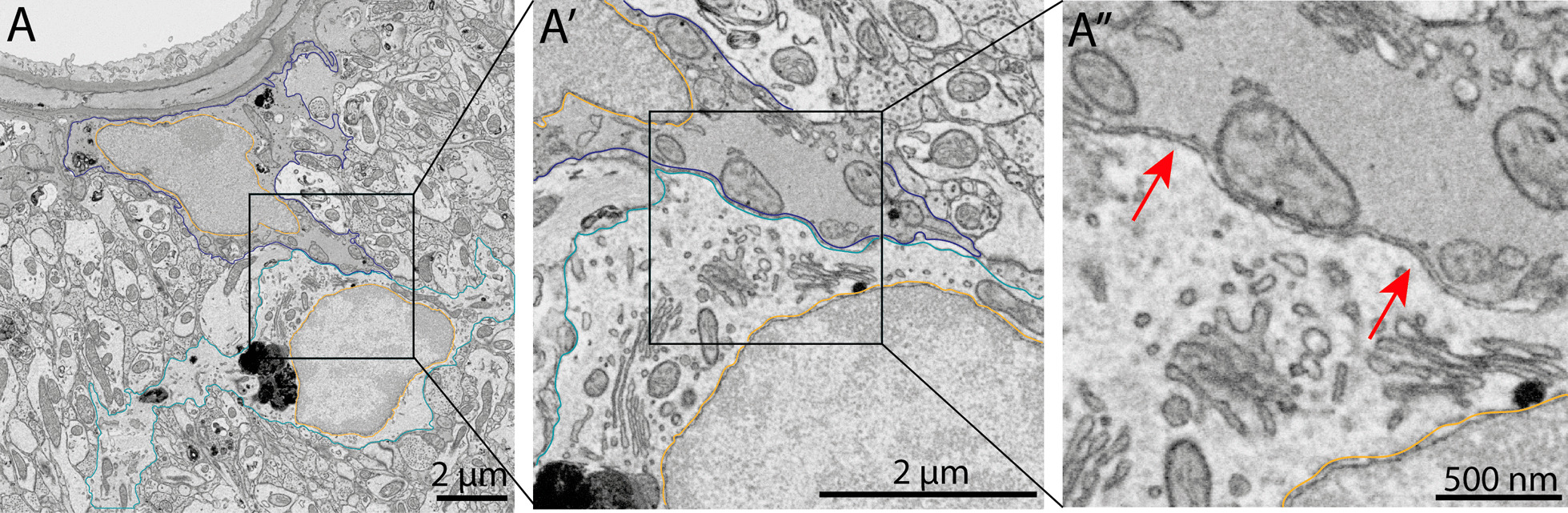
Blood vessel-associated dark astrocyte in the *stratum lacunosum-moleculare*. Representative 5 nm per pixel of resolution scanning electron microscopy images of dark astrocytes acquired in the ventral hippocampus CA1 *stratum lacunosum-moleculare* of 20-month-old APP-PS1 male mice. In **A**–**A″**, a dark astrocyte associated with the vasculature (pseudo-colored in red) is directly interacting with a typical microglial cell body. The red arrows in **A″** further indicate the close interaction between the two glial cells. Dark blue outline = dark astrocytic cytoplasm, light blue outline = microglial cytoplasm, yellow = nuclear membrane, red pseudo-coloring = blood vessel, red arrow = direct interaction between a microglial cell body and a dark astrocyte

Intriguingly, we often identified dark astrocytes next to a blood vessel interacting with other dark astrocytic cell bodies (Fig. 7C), typical astrocytic bodies (Fig. 7A), in addition to microglial cell bodies (Fig. 9A). While it is still unknown why dark astrocytes often come in close contact with microglial and astrocytic cell bodies, occupying satellite positions, typical astrocytes are known to interact with juxtavascular microglia [95, 124], in addition to contacting neighboring astrocytes notably through their complex and branched processes [119]. Overall, we examined the dark astrocytic state for the first time in adulthood, as well as in aged AD pathology, and found that it is characterized by the presence of glycogen granules, several markers of cellular stress, increased phagocytic capabilities (e.g., abundance of mature lysosomes and numerous phagosomes), a unique electron-dense cytoplasm and nucleoplasm, and a partial to total loss of the nuclear chromatin pattern.

### Dark astrocytes are observed in the hippocampal head of an aged human post-mortem brain sample

**Fig. 10:**
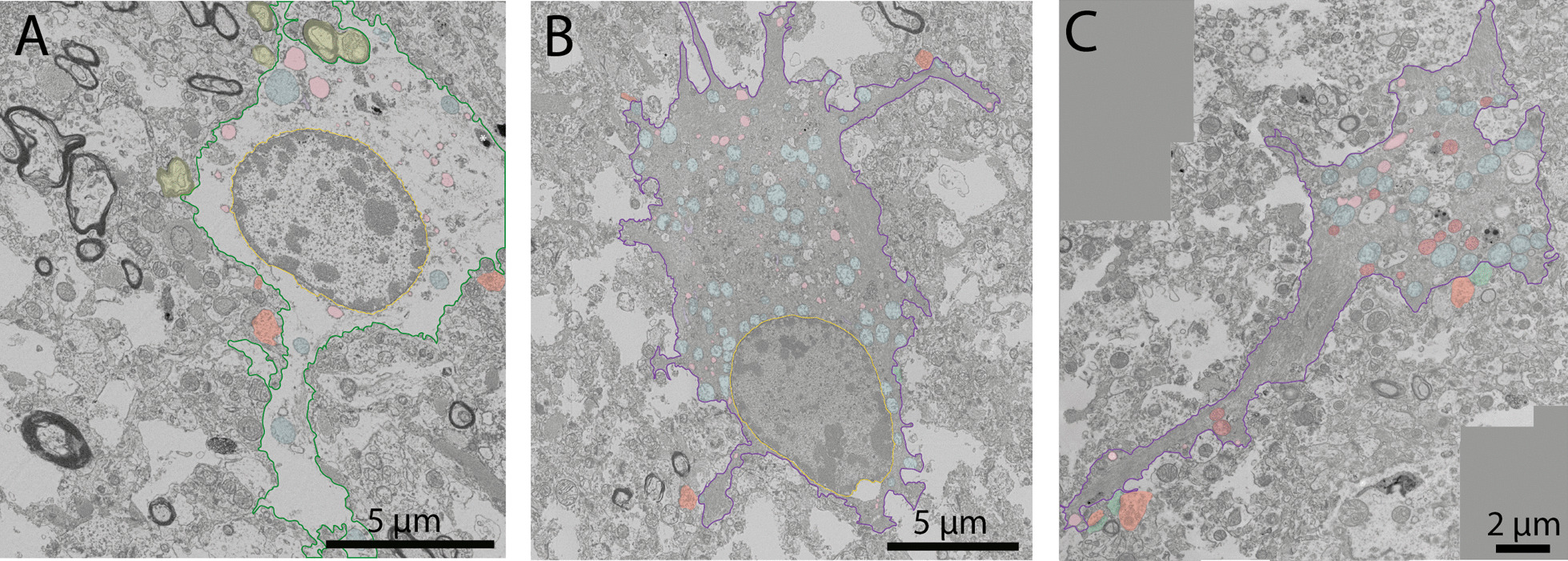
Typical and dark astrocytes in human post-mortem brain samples. Representative 5 nm per pixel of resolution scanning electron microscopy images of a typical (in **A**) and dark astrocytes (**B**, **C**) in the hippocampal head of an aged female (81-year-old, cause of death—asphyxia, post-mortem delay of 18 h). In **A**, a typical astrocyte interacts with several axon terminals (pseudo-colored in orange) and myelinated axons (pseudo-colored in yellow). The astrocyte possesses several fully digested phagosomes (pseudo-colored in pink) and altered mitochondria (pseudo-colored in blue). In **B** a dark astrocyte with several angular processes is making direct contacts with axon terminals (pseudo-colored in orange) and displaying several signs of cellular stress such as altered mitochondria (pseudo-colored in blue) and dilated endoplasmic reticulum (pseudo-colored in purple). In **C** a dark astrocytic process interacts with axon terminals (pseudo-colored in orange) and dendritic spine (pseudo-colored in green). The dark process contains several fully digested phagosomes (pseudo-colored in pink), altered mitochondria (pseudo-colored in blue), and healthy mitochondria (pseudo-colored in red). Yellow outline = nuclear membrane, green outline = typical astrocytic cytoplasm, purple outline = dark astrocytic cytoplasm, yellow pseudo-coloring = myelinated axons, orange pseudo-coloring = axon terminals, green pseudo-coloring = dendritic spines, blue pseudo-coloring = altered mitochondria, red pseudo-coloring = non-altered mitochondria, pink pseudo-coloring = fully digested phagosomes, purple pseudo-coloring = dilated endoplasmic reticulum

Previous studies revealed in the human post-mortem brain following brain injury and brain tumors the presence of a dark astrocytic state [61–64], much like the cells described in the spinal cord cultures of embryonic mice [67] and rat models of electroshock [66], kainic and pentylenetetrazole injection, as well as brain injury [65]. As we observed dark astrocytes in 3- to 4-month-old and 20-month-old C57BL/6J mice and APP-PS1 mice, we further investigated their conservation across species by examining an aged human post-mortem brain sample (female, 81 years old, post-mortem delay 18 h) in the hippocampal head, a region shown to have significant age-related atrophy [98, 100, 101]. Similar to what we uncovered in the mouse brain, we denoted the presence of dark astrocytes that possessed an electron-dense cytoplasm and nucleoplasm in this sample. To the best of our knowledge, this is the first case report that identifies and characterizes this dark astrocytic state among the human hippocampal head in the context of aging. The dark astrocytic cell bodies were seen contacting axon terminals, and their processes were interacting with numerous synapses (both axon terminals and dendritic spines at the same excitatory synapse). Much like typical astrocytes (Fig. 10A), the dark astrocytic cell bodies (Fig. 10B) and their processes (Fig. 10C) also possessed angular protuberances contacting the parenchymal elements and the vasculature. In addition, human dark astrocytes contained several altered mitochondria and dilated ER cisternae, ultrastructural markers of cellular stress which were also previously identified in non-dark astrocytes from human post-mortem parietal cortex samples of patients with AD [125] and in dark astrocytes from human post-mortem samples of brain injury and brain tumors [61–63]. Moreover, in the human dark astrocytes we have examined, several fully digested phagosomes were identified inside the cell body and processes.

## Discussion

Astrocytes which are notably involved in impaired glutamine synthesis, but beneficial for their ability to clear and degrade Aß, and phagocytose dystrophic neurites, were shown to be key players in AD pathology [38, 45, 46, 53, 55, 104, 126, 127]. While investigations of astrocytes in the pathogenesis of AD have gained traction in the last decade, few studies investigated their ultrastructure and to the best of the authors’ knowledge, this is the first quantification of astrocytic intracellular contents and parenchymal interactions by electron microscopy in an aged mouse model of AD pathology. As aging is the most predominant risk factor to developing AD [1], it is crucial to further explore the astrocytic ultrastructure in this context. In addition, as previous studies identified morphological and molecular heterogeneity of astrocytes based on their proximity to Aß plaques and dystrophic neurites [104–106], it is also important to take into account their location to AD hallmarks.

In the current study, we first performed an in situ ultrastructural investigation of typical astrocytes, notably based on their distance to Aß plaques/dystrophic neurites, in the ventral hippocampus CA1 *strata lacunosum-moleculare* and *radiatum* of 20-month-old APP-PS1 and age-matched C57BL/6J male mice. In a previous study, Sanchez-Mico et al*.* observed a decrease in phagolysosomal digestion of dystrophic neurites by astrocytes near Aß plaques in the hippocampus of 12-month-old APP751_sl_ mice, which was suggested to result from a reduced astrocytic expression of proteins associated with phagocytosis (Megf10, MerTK) [128]. In our aged mouse model of AD pathology, in the *stratum radiatum*, typical astrocytes contained more mature tertiary lysosomes but fewer primary lysosomes far from Aß plaques, indicating a shift in maturation of their lysosomal pathway. Yet, while the lysosomes shifted from an immature to a mature appearance, the numbers of fully and partially digested phagosomes within the astrocytic cytoplasm were relatively unchanged between groups.

Interestingly, typical astrocytes in the APP-PS1 possessed far more lipid bodies, a feature previously shown to protect neurons against neurotoxicity [129–132]. Indeed, several studies have demonstrated that neurons accumulate unstable lipotoxic elements in the presence of elevated levels of reactive oxygen species (ROS) and altered mitochondria, which are then shuttled to nearby glial cells. This was notably shown in primary mixed glial cells from the olfactory bulb of *Apoe*^−/−^ male mice, a model used to investigate the function of APOE, followed by an injection of rotenone to increase ROS levels, and in primary astrocytic cultures from ApoE knockout (KO) mice [129, 132]. In inflammatory conditions such as the chronic exposition to noradrenaline or hypoxic stress, primary astrocytic cultures from the neocortex of rats as well as organotypic brain slices from 2- to 4-month-old rats also presented a similar accumulation of lipid droplets, which was suggested to be associated with the protection of neurons from lipotoxicity [133].

We also found that typical astrocytes located in the *stratum radiatum* of APP-PS1 mice *vs* C57BL/6J mice interacted more with dendritic spines and axon terminals. Similarly, typical astrocytes near Aß plaques/dystrophic neurites in the *stratum lacunosum-moleculare* of aged APP-PS1 mice *vs* C57BL/6J mice contacted more synaptic elements, specifically dendritic spines. In AD pathology, astrocytes were previously reported to negatively influence synaptic numbers, notably via mechanisms that include complement-mediated phagocytosis of synaptic elements [134]. In 6-month-old 5XFAD mice which were exposed to contextual fear conditioning, astrocytes in the dentate gyrus showed a reduced colocalization between PSD95, a marker of post-synaptic density, and GFAP [135], which labels a subset of astrocytes, including ‘reactive’ ones [136, 137]. Synaptic loss near Aß plaques was also reduced in 7- to 13-month-old PS2APP mice, a model of AD pathology, crossed with mice KO for complement 3 (C3) [138], a molecule largely expressed by astrocytes [139]. Similarly, in the hippocampus of 16-month-old APP-PS1 C3 KO mice, levels of synaptic proteins (synapsin-1, synaptophysin, GluR1, PSD95 and Homer1) and pre- and post-synaptic puncta density (in the CA3 specifically, measured using staining for VGlut2 and GluR1, respectively) increased compared to APP-PS1 mice [140], highlighting the astrocytic impact on synaptic loss in AD pathology. As we observed an increase in phagosomes within astrocytes near Aß and dystrophic neurites in the *stratum lacunosum-moleculare*, it is a possibility that these astrocytes interact more with synaptic elements to phagocytose them. Future studies will be required to confirm this hypothesis, as well as investigate the impact of aging on astrocytic phagocytosis over the course of AD pathology. Another possible explanation for the increase in astrocyte–synapse interactions that we measured in the *stratum lacunosum-moleculare* of APP-PS1 mice could be the increase in the cytoplasmic perimeter of astrocytes near Aß plaques/dystrophic neurites compared to the ones far from these hallmarks, a morphological difference that was previously reported in the hippocampus of 6- compared to 18-month-old TgF344-AD rats as well as in the dentate gyrus and CA1 of 18-month-old 3xTg mice, both models of AD pathology [105, 106]. Indeed, morphological atrophy (observed far from Aß plaques) *vs* hypertrophy (in proximity to Aß plaques) was denoted in various brain regions (e.g., hippocampus, cerebral cortex) [104–106]. Future studies will be required, however, to determine the functional implications of these morphological changes.

Another interesting feature of typical astrocytes that we found near Aß plaques/dystrophic neurites is their accumulation of glycogen granules. Preferentially located in the astrocytic processes nearby synapses [110], glycogen granules were shown to be involved in learning and memory processes in 3-month-old C57Bl/6N male mice injected in the hippocampus with 1,4-dideoxy-1,4-imino-d-arabinitol, a glycogen phosphorylase inhibitor which blocks glycogenolysis [107, 108]. Both in humans and primates, glycogen accumulation was seen following reperfusion in ischemic stroke, where it was associated with a dysfunctional glycogenolytic pathway, the latter being responsible for the breaking down of glycogen [141]. This increase in glycogen granules was previously associated with the presence of intracellular Aß in astrocytes from post-mortem brain samples of AD patients [142]. This is in line with our observations which identified a high presence of glycogen granules specifically near fibrillar Aß plaques and dystrophic neurites. Astrocytic lactate was shown to be reduced in 6- to 7-month-old female 3xTg mice compared to age-matched controls and was associated with synaptic deficits [143]. A decrease in astrocytic TCA metabolites coupled with functional neuronal excitatory signaling alterations was also previously noted in slices from the hippocampus CA1 of 2- and 4-month-old 5xFAD male mice [144]. Therefore, investigating the glycolytic metabolism disturbances in astrocytes could help better understand their impact on the synaptic dysfunction observed across AD pathology.

The concept of glial heterogeneity, notably in neuropathological conditions such as AD, has gained momentum in recent years [55, 56, 145–156, 156–162]. An exponential number of studies using single-cell/nucleus RNA sequencing which aimed to identify unique molecular signatures of glial cells have come out, all pointing toward various clusters of glial cells up- and down-regulating specific gene signatures. Similar techniques were applied to elucidate the transcriptomic heterogeneity of astrocytes in AD pathology, both in mouse models and human post-mortem brain samples, notably identifying the disease-associated astrocytes in mouse models of AD pathology and the reactive astrocytic state in human post-mortem brain samples of patients with AD [55, 56, 159]. These studies have investigated the heterogeneity in astrocytic transcriptomic signatures, leaving an important gap in knowledge pertaining to their ultrastructural heterogeneity in AD pathology.

In our in situ investigation of astrocytic heterogeneity, we have identified a unique astrocytic state, the dark astrocytes, combining astrocytic identification criteria with similar ultrastructural features as the dark microglia previously identified in middle-aged and aged APP-PS1 male mice [59, 70]. Dark astrocytes were previously observed in rat models of electroshock [66], compressive and concussive head injury, pentylenetetrazole or kainic acid treatment [65], and spinal cord culture from embryonic mice [67]. Interestingly, unlike the findings of Gallyas et al. [66] and Tóth et al*.* [65] which did not observe dark astrocytes in control animals, we observed these electron-dense cells in young and aged C57BL/6J mice (3–4 and 20-month-old) as well as in aged APP-PS1 mice (20-month-old). However, future studies are warranted to quantify these cells over time and determine whether they become more abundant with aging and pathology. Interestingly, these cells have been observed in conditions associated with (neuro)inflammation, such as in AD pathology, as well as following kainic acid intraperitoneal injections and brain injury [65]. In-depth investigation analyzing the effect of the brain’s microenvironment on the appearance of dark astrocytes will be important to perform. Much like the previous observations of dark astrocytes in rodents [65–67] as well as dark microglia, a microglial state associated with an electron-dense cytoplasm and nucleoplasm [59, 60, 81, 163], we observed several signs of oxidative stress such as altered mitochondria, dilated ER and Golgi apparatus, in dark astrocytes.

Dark astrocytes were shown to internalize dystrophic neurites, highlighting a potential role for these cells in the pathogenesis of AD. This feature was also identified in typical astrocytes from 6- and 12-month-old APP-PS1 mice [53], alongside several pre- and post-synaptic elements and fibrillar Aß. A full quantification of their intracellular content will help determine if these cells phagocytose more or less of these elements compared to their typical counterparts. Indeed, Sanchez-Mico et al*.* demonstrated that Aß impaired the ability of astrocytes to phagocytose dystrophic synapses in the hippocampus of 12-month-old APP751_sl_ mice, a model of AD pathology [128]. It remains to be determined if dark astrocytes’ ability to phagocytose dystrophic neurites is also impaired in aged APP-PS1 mice and if this dysfunctional ability is conserved in human post-mortem brain samples.

We further observed the presence of dark astrocytes in the hippocampal head of an aged individual, similar to dark astrocytes in the cerebral cortex of male and female post-mortem samples of brain injury and brain tumors [61–63], as well as in brain samples of male and female patients with hemangioblastoma [64]. These dark astrocytes, much like the ones uncovered in mice, possessed signs of cellular stress (altered mitochondria and dilated ER). This conservation, of both the electron-dense state and the oxidative stress markers, across species, denotes similarities between mice and humans: uncovering the mechanism behind the appearance of the dark astrocytes and their function would be key to better understand the diverse, contextually dependent astrocytic response to aging and AD pathology.

## Conclusion

We investigated in situ using nanoscale-resolution SEM the ultrastructural alterations in cellular contents and parenchymal interactions of typical astrocytes in aged APP-PS1 and age-matched C57BL/6J male mice. In both examined layers of the hippocampus, we observed increased interactions with synaptic elements along with increased signs of phagolysosomal activity, identifying astrocytic changes linked to AD pathology and their proximity to Aß plaques. Moreover, this ultrastructural study examining astrocytic heterogeneity in aging and AD pathology further characterized a unique astrocytic state, the dark astrocytes, in mice and human post-mortem brain samples. The dark astrocytes displayed markers of cellular stress (e.g., dilated ER and Golgi apparatus), internalized dystrophic neurites (in aged APP-PS1 mice), accumulated glycogen granules within their cytoplasm, and were often located near the vasculature. In addition, we confirmed the conservation of this state in aged human post-mortem brain samples, more specifically among the hippocampal head, highlighting key similarities between species. In short, this study underlines novel ultrastructural alterations of astrocytes in the hippocampus of aged AD pathology, while identifying a dark astrocytic state both in mice and humans.

## Supplementary Information

**Additional file 1: Figure S1.** A blood vessel-associated dark astrocyte imaged serially distances displays several phagosomes. In A–A″, pictures of a dark astrocyte taken serially at a distance of 5–6 µm were acquired. The dark astrocytic cell body, associated with a blood vessel, shows numerous contacts with dendritic spines (pseudo-colored in orange) and axon terminals (pseudo-colored in blue) and contains several partially digested phagosomes (pseudo-colored in purple), notably axon terminals and dendritic spines. Yellow = nuclear membrane, red pseudo-coloring = blood vessel, orange pseudo-coloring = dendritic spine, blue pseudo-coloring = axon terminals, green pseudo-coloring = mitochondria, purple pseudo-coloring = partially digested phagosomes

## Data Availability

All data presented in this study are available from the corresponding author upon reasonable request.

## Abbreviations

+: Positive
Aß: Amyloid beta
AD: Alzheimer’s disease
AR: Aspect ratio
CA: *Cornu* *ammonis*
C3: Complement 3
ER: Endoplasmic reticulum
GFAP: Glial fibrillary acidic protein
KO: Knock-out
NFT: Neurofibrillary tangles
PB: Phosphate buffer
PBS: Phosphate-buffered saline
PBS-T: Phosphate-buffered saline with Triton
PFA: Paraformaldehyde
ROS: Reactive oxygen species
RT: Room temperature
SEM: Scanning electron microscope
TB: Tris buffer
TBS: Tris-buffered saline
TCA: Tricarboxylic acid cycle

## References

[1] Guerreiro R, Bras J (2015). The age factor in Alzheimer’s disease. Genome Med.

[2] Spires-Jones TL, Hyman BT (2014). The intersection of amyloid beta and tau at synapses in Alzheimer’s disease. Neuron.

[3] Halliday G (2017). Pathology and hippocampal atrophy in Alzheimer’s disease. Lancet Neurol.

[4] Marino S, Bonanno L, Lo Buono V, Ciurleo R, Corallo F, Morabito R (2019). Longitudinal analysis of brain atrophy in Alzheimer’s disease and frontotemporal dementia. J Int Med Res.

[5] Pini L, Pievani M, Bocchetta M, Altomare D, Bosco P, Cavedo E (2016). Brain atrophy in Alzheimer’s disease and aging. Ageing Res Rev.

[6] Terry RD, Masliah E, Salmon DP, Butters N, DeTeresa R, Hill R (1991). Physical basis of cognitive alterations in Alzheimer’s disease: synapse loss is the major correlate of cognitive impairment. Ann Neurol.

[7] Varma VR, Oommen AM, Varma S, Casanova R, An Y, Andrews RM (2018). Brain and blood metabolite signatures of pathology and progression in Alzheimer disease: a targeted metabolomics study. PLoS Med.

[8] Toledo JB, Arnold M, Kastenmüuller G, Chang R, Baillie RA, Han X (2017). Metabolic network failures in Alzheimer’s disease: a biochemical road map. Alzheimers Dement.

[9] Trushina E, Dutta T, Persson XMT, Mielke MM, Petersen RC (2013). Identification of altered metabolic pathways in plasma and CSF in mild cognitive impairment and Alzheimer’s disease using metabolomics. PLoS ONE.

[10] Costa AC, Joaquim HPG, Forlenza OV, Gattaz WF, Talib LL (2020). Three plasma metabolites in elderly patients differentiate mild cognitive impairment and Alzheimer’s disease: a pilot study. Eur Arch Psychiatry Clin Neurosci.

[11] Herholz K (2010). Cerebral glucose metabolism in preclinical and prodromal Alzheimer’s disease. Expert Rev Neurother.

[12] van der Velpen V, Teav T, Gallart-Ayala H, Mehl F, Konz I, Clark C (2019). Systemic and central nervous system metabolic alterations in Alzheimer’s disease. Alzheimers Res Ther.

[13] DeTure MA, Dickson DW (2019). The neuropathological diagnosis of Alzheimer’s disease. Mol Neurodegener.

[14] Mrdjen D, Fox EJ, Bukhari SA, Montine KS, Bendall SC, Montine TJ (2019). The basis of cellular and regional vulnerability in Alzheimer’s disease. Acta Neuropathol.

[15] Fanselow MS, Dong HW (2010). Are the dorsal and ventral hippocampus functionally distinct structures?. Neuron.

[16] Lee AR, Kim JH, Cho E, Kim M, Park M (2017). Dorsal and ventral hippocampus differentiate in functional pathways and differentially associate with neurological disease-related genes during postnatal development. Front Mol Neurosci.

[17] Masurkar AV (2018). Towards a circuit-level understanding of hippocampal CA1 dysfunction in Alzheimer’s disease across anatomical axes. J Alzheimers Dis Parkinsonism.

[18] Su L, Hayes L, Soteriades S, Williams G, Brain SA, Firbank MJ (2018). Hippocampal stratum radiatum, lacunosum and moleculare sparing in mild cognitive impairment. J Alzheimers Dis.

[19] Shaw K, Bell L, Boyd K, Grijseels DM, Clarke D, Bonnar O (2021). Neurovascular coupling and oxygenation are decreased in hippocampus compared to neocortex because of microvascular differences. Nat Commun.

[20] Herculano-Houzel S (2014). The glia/neuron ratio: how it varies uniformly across brain structures and species and what that means for brain physiology and evolution. Glia.

[21] Verkhratsky A, Butt AM (2018). The history of the decline and fall of the glial numbers legend. Neuroglia.

[22] Akdemir ES, Huang AYS, Deneen B (2020). Astrocytogenesis: where, when, and how. F1000Res.

[23] Şovrea AS, Boşca AB (2013). Astrocytes reassessment—an evolving concept part one: embryology, biology, morphology and reactivity. J Mol Psychiatry..

[24] Nahirney PC, Tremblay ME (2021). Brain ultrastructure: putting the pieces together. Front Cell Dev Biol.

[25] Wang F, Xu S, Pan F, Verkhratsky A, Huang JH (2022). Editorial: Natural products and brain energy metabolism: astrocytes in neurodegenerative diseases. Front Pharmacol.

[26] Mestre H, Mori Y, Nedergaard M (2020). The brain’s glymphatic system: current controversies. Trends Neurosci.

[27] Louveau A, Smirnov I, Keyes TJ, Eccles JD, Rouhani SJ, Peske JD (2015). Structural and functional features of central nervous system lymphatics. Nature.

[28] Iliff JJ, Lee H, Yu M, Feng T, Logan J, Nedergaard M (2013). Brain-wide pathway for waste clearance captured by contrast-enhanced MRI. J Clin Invest.

[29] Mulligan SJ, MacVicar BA (2004). Calcium transients in astrocyte endfeet cause cerebrovascular constrictions. Nature.

[30] Zonta M, Angulo MC, Gobbo S, Rosengarten B, Hossmann KA, Pozzan T (2003). Neuron-to-astrocyte signaling is central to the dynamic control of brain microcirculation. Nat Neurosci.

[31] Gordon GRJ, Mulligan SJ, MacVicar BA (2007). Astrocyte control of the cerebrovasculature. Glia.

[32] Verkhratsky A, Parpura V, Li B, Scuderi C (2021). Astrocytes: the housekeepers and guardians of the CNS. Adv Neurobiol.

[33] Ullian EM, Sapperstein SK, Christopherson KS, Barres BA (2001). Control of synapse number by glia. Science.

[34] Barker AJ, Koch SM, Reed J, Barres BA, Ullian EM (2008). Developmental control of synaptic receptivity. J Neurosci.

[35] Hama H, Hara C, Yamaguchi K, Miyawaki A (2004). PKC signaling mediates global enhancement of excitatory synaptogenesis in neurons triggered by local contact with astrocytes. Neuron.

[36] Augusto-Oliveira M, Arrifano GP, Takeda PY, Lopes-Araújo A, Santos-Sacramento L, Anthony DC (2020). Astroglia-specific contributions to the regulation of synapses, cognition and behaviour. Neurosci Biobehav Rev.

[37] Chung WS, Allen NJ, Eroglu C (2015). Astrocytes control synapse formation, function, and elimination. Cold Spring Harb Perspect Biol.

[38] Andersen JV, Christensen SK, Westi EW, Diaz-delCastillo M, Tanila H, Schousboe A (2021). Deficient astrocyte metabolism impairs glutamine synthesis and neurotransmitter homeostasis in a mouse model of Alzheimer’s disease. Neurobiol Dis.

[39] Verkhratsky A, Nedergaard M (2018). Physiology of astroglia. Physiol Rev.

[40] Tsacopoulos M, Magistretti PJ (1996). Metabolic coupling between glia and neurons. J Neurosci.

[41] Pellerin L, Bouzier-Sore AK, Aubert A, Serres S, Merle M, Costalat R (2007). Activity-dependent regulation of energy metabolism by astrocytes: an update. Glia.

[42] Wang Z, Zhang Q, Lin JR, Jabalameli MR, Mitra J, Nguyen N (2021). Deep post-GWAS analysis identifies potential risk genes and risk variants for Alzheimer’s disease, providing new insights into its disease mechanisms. Sci Rep.

[43] Smith AM, Davey K, Tsartsalis S, Khozoie C, Fancy N, Tang SS (2022). Diverse human astrocyte and microglial transcriptional responses to Alzheimer’s pathology. Acta Neuropathol.

[44] St-Pierre MK, VanderZwaag J, Loewen S, Tremblay MÈ (2022). All roads lead to heterogeneity: the complex involvement of astrocytes and microglia in the pathogenesis of Alzheimer’s disease. Front Cell Neurosci.

[45] Katsouri L, Birch AM, Renziehausen AWJ, Zach C, Aman Y, Steeds H (2020). Ablation of reactive astrocytes exacerbates disease pathology in a model of Alzheimer’s disease. Glia.

[46] Davis N, Mota BC, Stead L, Palmer EOC, Lombardero L, Rodríguez-Puertas R (2021). Pharmacological ablation of astrocytes reduces Aβ degradation and synaptic connectivity in an ex vivo model of Alzheimer’s disease. J Neuroinflammation.

[47] Apelt J, Ach K, Schliebs R (2003). Aging-related down-regulation of neprilysin, a putative β-amyloid-degrading enzyme, in transgenic Tg2576 Alzheimer-like mouse brain is accompanied by an astroglial upregulation in the vicinity of β-amyloid plaques. Neurosci Lett.

[48] Yamamoto N, Nakazawa M, Nunono N, Yoshida N, Obuchi A, Tanida M (2021). Protein kinases A and C regulate amyloid-β degradation by modulating protein levels of neprilysin and insulin-degrading enzyme in astrocytes. Neurosci Res.

[49] Yamamoto N, Ishikuro R, Tanida M, Suzuki K, Ikeda-Matsuo Y, Sobue K (2018). Insulin-signaling pathway regulates the degradation of amyloid β-protein via astrocytes. Neuroscience.

[50] Norton L, Shannon C, Gastaldelli A, DeFronzo RA (2022). Insulin: the master regulator of glucose metabolism. Metabolism.

[51] Wegiel J, Wang KC, Tarnawski M, Lach B (2000). Microglia cells are the driving force in fibrillar plaque formation, whereas astrocytes are a leading factor in plague degradation. Acta Neuropathol.

[52] Wisniewski HM, Wegiel J (1991). Spatial relationships between astrocytes and classical plaque components. Neurobiol Aging.

[53] Gomez-Arboledas A, Davila JC, Sanchez-Mejias E, Navarro V, Nuñez-Diaz C, Sanchez-Varo R (2018). Phagocytic clearance of presynaptic dystrophies by reactive astrocytes in Alzheimer’s disease. Glia.

[54] Serrano-Pozo A, Muzikansky A, Gómez-Isla T, Growdon JH, Betensky RA, Frosch MP (2013). Differential relationships of reactive astrocytes and microglia to fibrillar amyloid deposits in Alzheimer disease. J Neuropathol Exp Neurol.

[55] Habib N, McCabe C, Medina S, Varshavsky M, Kitsberg D, Dvir-Szternfeld R (2020). Disease-associated astrocytes in Alzheimer’s disease and aging. Nat Neurosci.

[56] Morabito S, Miyoshi E, Michael N, Shahin S, Martini AC, Head E (2021). Single-nucleus chromatin accessibility and transcriptomic characterization of Alzheimer’s disease. Nat Genet.

[57] Muñoz-Castro C, Noori A, Magdamo CG, Li Z, Marks JD, Frosch MP (2022). Cyclic multiplex fluorescent immunohistochemistry and machine learning reveal distinct states of astrocytes and microglia in normal aging and Alzheimer’s disease. J Neuroinflammation.

[58] Cabinio M, Saresella M, Piancone F, LaRosa F, Marventano I, Guerini FR (2018). Association between hippocampal shape, neuroinflammation, and cognitive decline in Alzheimer’s disease. J Alzheimers Dis.

[59] Bisht K, Sharma KP, Lecours C, Gabriela Sánchez M, El Hajj H, Milior G (2016). Dark microglia: a new phenotype predominantly associated with pathological states. Glia.

[60] St-Pierre MK, Carrier M, Lau V, Tremblay MÈ (2022). Investigating microglial ultrastructural alterations and intimate relationships with neuronal stress, dystrophy, and degeneration in mouse models of Alzheimer’s disease. Methods Mol Biol.

[61] Castejón OJ (2015). Biopathology of astrocytes in human traumatic and complicated brain injuries. Review and hypothesis. Folia Neuropathol.

[62] Castejón O (1999). Astrocyte subtypes in the gray matter of injured human cerebral cortex: a transmission electron microscope study. Brain Inj.

[63] Castejón OJ (1998). Morphological astrocytic changes in complicated human brain trauma. A light and electron microscopic study. Brain Inj.

[64] Shimura T, Hirano A, Llena JF (1985). Ultrastructure of cerebellar hemangioblastoma. Some new observations on the stromal cells. Acta Neuropathol.

[65] Tóth Z, Séress L, Tóth P, Ribak CE, Gallyas F (1997). A common morphological response of astrocytes to various injuries: “dark” astrocytes. A light and electron microscopic analysis. J Hirnforsch.

[66] Gallyas F, Horváth Z, Dávid K, Liposits Z (1994). An immediate morphopathologic response of a subpopulation of astrocytes to electroshock: “dark” astrocytes. Neurobiology (Bp).

[67] Munoz-Garcia D, Ludwin SK (1986). Gliogenesis in organotypic tissue culture of the spinal cord of the embryonic mouse. I. Immunocytochemical and ultrastructural studies. J Neurocytol.

[68] Hol EM, Pekny M (2015). Glial fibrillary acidic protein (GFAP) and the astrocyte intermediate filament system in diseases of the central nervous system. Curr Opin Cell Biol.

[69] Borchelt DR, Ratovitski T, van Lare J, Lee MK, Gonzales V, Jenkins NA (1997). Accelerated amyloid deposition in the brains of transgenic mice coexpressing mutant presenilin 1 and amyloid precursor proteins. Neuron.

[70] St-Pierre MK, Carrier M, González Ibáñez F, Šimončičová E, Wallman MJ, Vallières L (2022). Ultrastructural characterization of dark microglia during aging in a mouse model of Alzheimer’s disease pathology and in human post-mortem brain samples. J Neuroinflammation.

[71] El Hajj H, Savage JC, Bisht K, Parent M, Vallières L, Rivest S (2019). Ultrastructural evidence of microglial heterogeneity in Alzheimer’s disease amyloid pathology. J Neuroinflammation.

[72] Bisht K, El Hajj H, Savage JC, Sánchez MG, Tremblay MÈ (2016). Correlative light and electron microscopy to study microglial interactions with β-amyloid plaques. J Vis Exp.

[73] Paxinos G, Franklin KBJ (2012). Paxinos and Franklin’s the Mouse brain in stereotaxic coordinates.

[74] Peters A, Palay SL, Webster H (1991). The fine structure of the nervous system: neurons and their supporting cells.

[75] Turmaine M, Raza A, Mahal A, Mangiarini L, Bates GP, Davies SW (2000). Nonapoptotic neurodegeneration in a transgenic mouse model of Huntington’s disease. Proc Natl Acad Sci USA.

[76] Kherani ZS, Auer RN (2008). Pharmacologic analysis of the mechanism of dark neuron production in cerebral cortex. Acta Neuropathol.

[77] Colbourne F, Sutherland GR, Auer RN (1999). Electron microscopic evidence against apoptosis as the mechanism of neuronal death in global ischemia. J Neurosci.

[78] Dietrich WD, Alonso O, Halley M, Busto R (1996). Delayed posttraumatic brain hyperthermia worsens outcome after fluid percussion brain injury: a light and electron microscopic study in rats. Neurosurgery.

[79] Kuroiwa T, Nagaoka T, Ueki M, Yamada I, Miyasaka N, Akimoto H (1998). Different apparent diffusion coefficient: water content correlations of gray and white matter during early ischemia. Stroke.

[80] St-Pierre MK, Bordeleau M, Tremblay MÈ (2019). Visualizing Dark Microglia. Methods Mol Biol.

[81] St-Pierre MK, Šimončičová E, Bögi E, Tremblay MÈ (2020). Shedding light on the dark side of the microglia. ASN Neuro.

[82] Bordeleau M, Lacabanne C, Fernández de Cossío L, Vernoux N, Savage JC, González-Ibáñez F (2020). Microglial and peripheral immune priming is partially sexually dimorphic in adolescent mouse offspring exposed to maternal high-fat diet. J Neuroinflammation.

[83] Tremblay MÈ, Majewska AK (2019). Ultrastructural analyses of microglial interactions with synapses. Methods Mol Biol.

[84] Bordeleau M, Fernández de Cossío L, Lacabanne C, Savage JC, Vernoux N, Chakravarty M (2021). Maternal high-fat diet modifies myelin organization, microglial interactions, and results in social memory and sensorimotor gating deficits in adolescent mouse offspring. Brain Behav Immun Health..

[85] Hui CW, St-Pierre MK, Detuncq J, Aumailley L, Dubois MJ, Couture V (2018). Nonfunctional mutant Wrn protein leads to neurological deficits, neuronal stress, microglial alteration, and immune imbalance in a mouse model of Werner syndrome. Brain Behav Immun.

[86] Miyazono Y, Hirashima S, Ishihara N, Kusukawa J, Nakamura KI, Ohta K (2018). Uncoupled mitochondria quickly shorten along their long axis to form indented spheroids, instead of rings, in a fission-independent manner. Sci Rep.

[87] Decoeur F, Picard K, St-Pierre MK, Greenhalgh AD, Delpech JC, Sere A (2022). N-3 PUFA deficiency affects the ultrastructural organization and density of white matter microglia in the developing brain of male mice. Front Cell Neurosci.

[88] Prats C, Graham TE, Shearer J (2018). The dynamic life of the glycogen granule. J Biol Chem.

[89] Versaevel M, Braquenier JB, Riaz M, Grevesse T, Lantoine J, Gabriele S (2014). Super-resolution microscopy reveals LINC complex recruitment at nuclear indentation sites. Sci Rep.

[90] Henry MS, Bisht K, Vernoux N, Gendron L, Torres-Berrio A, Drolet G (2018). Delta opioid receptor signaling promotes resilience to stress under the repeated social defeat paradigm in mice. Front Mol Neurosci.

[91] Hart ML, Lauer JC, Selig M, Hanak M, Walters B, Rolauffs B (2018). Shaping the cell and the future: recent advancements in biophysical aspects relevant to regenerative medicine. J Funct Morphol Kinesiol.

[92] Leyh J, Paeschke S, Mages B, Michalski D, Nowicki M, Bechmann I (2021). Classification of microglial morphological phenotypes using machine learning. Front Cell Neurosci.

[93] Hui CW, St-Pierre A, El Hajj H, Remy Y, Hébert SS, Luheshi GN (2018). Prenatal immune challenge in mice leads to partly sex-dependent behavioral, microglial, and molecular abnormalities associated with Schizophrenia. Front Mol Neurosci.

[94] Lecours C, St-Pierre MK, Picard K, Bordeleau M, Bourque M, Awogbindin IO (2020). Levodopa partially rescues microglial numerical, morphological, and phagolysosomal alterations in a monkey model of Parkinson’s disease. Brain Behav Immun.

[95] Mondo E, Becker SC, Kautzman AG, Schifferer M, Baer CE, Chen J (2020). A developmental analysis of juxtavascular microglia dynamics and interactions with the vasculature. J Neurosci.

[96] Savage JC, St-Pierre MK, Carrier M, El Hajj H, Novak SW, Sanchez MG (2020). Microglial physiological properties and interactions with synapses are altered at presymptomatic stages in a mouse model of Huntington’s disease pathology. J Neuroinflammation.

[97] Yasumoto Y, Stoiljkovic M, Kim JD, Sestan-Pesa M, Gao XB, Diano S (2021). Ucp2-dependent microglia-neuronal coupling controls ventral hippocampal circuit function and anxiety-like behavior. Mol Psychiatry.

[98] Malykhin NV, Bouchard TP, Camicioli R, Coupland NJ (2008). Aging hippocampus and amygdala. NeuroReport.

[99] Russo ML, Molina-Campos E, Ybarra N, Rogalsky AE, Musial TF, Jimenez V (2021). Variability in sub-threshold signaling linked to Alzheimer’s disease emerges with age and amyloid plaque deposition in mouse ventral CA1 pyramidal neurons. Neurobiol Aging.

[100] Veldsman M, Nobis L, Alfaro-Almagro F, Manohar S, Husain M (2021). The human hippocampus and its subfield volumes across age, sex and APOE e4 status. Brain Commun.

[101] Driscoll I, Hamilton DA, Petropoulos H, Yeo RA, Brooks WM, Baumgartner RN (2003). The aging hippocampus: cognitive, biochemical and structural findings. Cereb Cortex.

[102] Schitine C, Nogaroli L, Costa MR, Hedin-Pereira C (2015). Astrocyte heterogeneity in the brain: from development to disease. Front Cell Neurosci.

[103] Zhou B, Zuo YX, Jiang RT (2019). Astrocyte morphology: diversity, plasticity, and role in neurological diseases. CNS Neurosci Ther.

[104] Li KY, Gong PF, Li JT, Xu NJ, Qin S (2020). Morphological and molecular alterations of reactive astrocytes without proliferation in cerebral cortex of an APP/PS1 transgenic mouse model and Alzheimer’s patients. Glia.

[105] Olabarria M, Noristani HN, Verkhratsky A, Rodríguez JJ (2010). Concomitant astroglial atrophy and astrogliosis in a triple transgenic animal model of Alzheimer’s disease. Glia.

[106] Mampay M, Velasco-Estevez M, Rolle SO, Chaney AM, Boutin H, Dev KK (2020). Spatiotemporal immunolocalisation of REST in the brain of healthy ageing and Alzheimer’s disease rats. FEBS Open Bio.

[107] Vezzoli E, Calì C, De Roo M, Ponzoni L, Sogne E, Gagnon N (2020). Ultrastructural evidence for a role of astrocytes and glycogen-derived lactate in learning-dependent synaptic stabilization. Cereb Cortex.

[108] Alberini CM, Cruz E, Descalzi G, Bessières B, Gao V (2018). Astrocyte glycogen and lactate: new insights into learning and memory mechanisms. Glia.

[109] Gertz HJ, Cervos-Navarro J, Frydl V, Schultz F (1985). Glycogen accumulation of the aging human brain. Mech Ageing Dev.

[110] Calì C, Tauffenberger A, Magistretti P (2019). The strategic location of glycogen and lactate: from body energy reserve to brain plasticity. Front Cell Neurosci.

[111] Mohammed H, Al-Awami AK, Beyer J, Cali C, Magistretti P, Pfister H (2018). Abstractocyte: a visual tool for exploring nanoscale astroglial cells. IEEE Trans Vis Comput Graph.

[112] Calì C, Baghabra J, Boges DJ, Holst GR, Kreshuk A, Hamprecht FA (2016). Three-dimensional immersive virtual reality for studying cellular compartments in 3D models from EM preparations of neural tissues. J Comp Neurol.

[113] Apátiga-Pérez R, Soto-Rojas LO, Campa-Córdoba BB, Luna-Viramontes NI, Cuevas E, Villanueva-Fierro I (2022). Neurovascular dysfunction and vascular amyloid accumulation as early events in Alzheimer’s disease. Metab Brain Dis.

[114] Farkas E, Luiten PG (2001). Cerebral microvascular pathology in aging and Alzheimer’s disease. Prog Neurobiol.

[115] Shabir O, Berwick J, Francis SE (2018). Neurovascular dysfunction in vascular dementia, Alzheimer’s and atherosclerosis. BMC Neurosci.

[116] Klohs J (2019). An integrated view on vascular dysfunction in Alzheimer’s disease. Neurodegener Dis.

[117] Solis E, Hascup KN, Hascup ER (2020). Alzheimer’s disease: the link between amyloid-β and neurovascular dysfunction. J Alzheimers Dis.

[118] Calì C, Agus M, Kare K, Boges DJ, Lehväslaiho H, Hadwiger M (2019). 3D cellular reconstruction of cortical glia and parenchymal morphometric analysis from serial block-face electron microscopy of juvenile rat. Prog Neurobiol.

[119] Aten S, Kiyoshi CM, Arzola EP, Patterson JA, Taylor AT, Du Y (2022). Ultrastructural view of astrocyte arborization, astrocyte–astrocyte and astrocyte–synapse contacts, intracellular vesicle-like structures, and mitochondrial network. Prog Neurobiol.

[120] Nagy JI, Rash JE (2003). Astrocyte and oligodendrocyte connexins of the glial syncytium in relation to astrocyte anatomical domains and spatial buffering. Cell Commun Adhes.

[121] Quigley HA (1977). Gap junctions between optic nerve head astrocytes. Invest Ophthalmol Vis Sci.

[122] Quillen S, Schaub J, Quigley H, Pease M, Korneva A, Kimball E (2020). Astrocyte responses to experimental glaucoma in mouse optic nerve head. PLoS ONE.

[123] Bisht K, Sharma K, Lacoste B, Tremblay MÈ (2016). Dark microglia: why are they dark?. Commun Integr Biol..

[124] Joost E, Jordão MJC, Mages B, Prinz M, Bechmann I, Krueger M (2019). Microglia contribute to the glia limitans around arteries, capillaries and veins under physiological conditions, in a model of neuroinflammation and in human brain tissue. Brain Struct Funct.

[125] Baloyannis SJ, Larrivee D (2019). Mitochondria and Alzheimer’s disease an electron microscopy study. Redirecting Alzheimer strategy.

[126] Verkhratsky A, Rodrigues JJ, Pivoriunas A, Zorec R, Semyanov A (2019). Astroglial atrophy in Alzheimer’s disease. Pflugers Arch.

[127] Spanos F, Liddelow SA (2020). An overview of astrocyte responses in genetically induced Alzheimer’s disease mouse models. Cells.

[128] Sanchez-Mico MV, Jimenez S, Gomez-Arboledas A, Muñoz-Castro C, Romero-Molina C, Navarro V (2021). Amyloid-β impairs the phagocytosis of dystrophic synapses by astrocytes in Alzheimer’s disease. Glia.

[129] Liu L, MacKenzie KR, Putluri N, Maletić-Savatić M, Bellen HJ (2017). The glia-neuron lactate shuttle and elevated ROS promote lipid synthesis in neurons and lipid droplet accumulation in glia via APOE/D. Cell Metab.

[130] Liu L, Zhang K, Sandoval H, Yamamoto S, Jaiswal M, Sanz E (2015). Glial lipid droplets and ROS induced by mitochondrial defects promote neurodegeneration. Cell.

[131] Moulton MJ, Barish S, Ralhan I, Chang J, Goodman LD, Harland JG (2021). Neuronal ROS-induced glial lipid droplet formation is altered by loss of Alzheimer’s disease-associated genes. Proc Natl Acad Sci USA.

[132] Ioannou MS, Jackson J, Sheu SH, Chang CL, Weigel AV, Liu H (2019). Neuron-astrocyte metabolic coupling protects against activity-induced fatty acid toxicity. Cell.

[133] Smolič T, Tavčar P, Horvat A, Černe U, Halužan Vasle A, Tratnjek L (2021). Astrocytes in stress accumulate lipid droplets. Glia.

[134] Hulshof LA, van Nuijs D, Hol EM, Middeldorp J (2022). The role of astrocytes in synapse loss in Alzheimer’s disease: a systematic review. Front Cell Neurosci.

[135] Choi M, Lee SM, Kim D, Im HI, Kim HS, Jeong YH (2021). Disruption of the astrocyte–neuron interaction is responsible for the impairments in learning and memory in 5XFAD mice: an Alzheimer’s disease animal model. Mol Brain.

[136] Xu J (2018). New insights into GFAP negative astrocytes in calbindin D28k immunoreactive astrocytes. Brain Sci.

[137] Tatsumi K, Isonishi A, Yamasaki M, Kawabe Y, Morita-Takemura S, Nakahara K (2018). Olig2-lineage astrocytes: a distinct subtype of astrocytes that differs from GFAP astrocytes. Front Neuroanat.

[138] Wu T, Dejanovic B, Gandham VD, Gogineni A, Edmonds R, Schauer S (2019). Complement C3 is activated in human AD brain and is required for neurodegeneration in mouse models of amyloidosis and tauopathy. Cell Rep.

[139] Lian H, Litvinchuk A, Chiang ACA, Aithmitti N, Jankowsky JL, Zheng H (2016). Astrocyte-microglia cross talk through complement activation modulates amyloid pathology in mouse models of Alzheimer’s disease. J Neurosci.

[140] Shi Q, Chowdhury S, Ma R, Le KX, Hong S, Caldarone BJ (2017). Complement C3 deficiency protects against neurodegeneration in aged plaque-rich APP/PS1 mice. Sci Transl Med..

[141] Cai Y, Guo H, Fan Z, Zhang X, Wu D, Tang W (2020). Glycogenolysis is crucial for astrocytic glycogen accumulation and brain damage after reperfusion in ischemic stroke. Science.

[142] Kurt MA, Davies DC, Kidd M (1999). β-amyloid immunoreactivity in astrocytes in Alzheimer’s disease brain biopsies: an electron microscope study. Exp Neurol.

[143] Le Douce J, Maugard M, Veran J, Matos M, Jégo P, Vigneron PA (2020). Impairment of glycolysis-derived l-serine production in astrocytes contributes to cognitive deficits in Alzheimer’s disease. Cell Metab.

[144] Andersen JV, Skotte NH, Christensen SK, Polli FS, Shabani M, Markussen KH (2021). Hippocampal disruptions of synaptic and astrocyte metabolism are primary events of early amyloid pathology in the 5xFAD mouse model of Alzheimer’s disease. Cell Death Dis.

[145] Hu Y, Fryatt GL, Ghorbani M, Obst J, Menassa DA, Martin-Estebane M (2021). Replicative senescence dictates the emergence of disease-associated microglia and contributes to Aβ pathology. Cell Rep.

[146] Krasemann S, Madore C, Cialic R, Baufeld C, Calcagno N, El Fatimy R (2017). The TREM2-APOE pathway drives the transcriptional phenotype of dysfunctional microglia in neurodegenerative diseases. Immunity.

[147] Clayton K, Delpech JC, Herron S, Iwahara N, Ericsson M, Saito T (2021). Plaque associated microglia hyper-secrete extracellular vesicles and accelerate tau propagation in a humanized APP mouse model. Mol Neurodegener.

[148] Srinivasan K, Friedman BA, Etxeberria A, Huntley MA, van der Brug MP, Foreman O (2020). Alzheimer’s patient microglia exhibit enhanced aging and unique transcriptional activation. Cell Rep.

[149] Keren-Shaul H, Spinrad A, Weiner A, Matcovitch-Natan O, Dvir-Szternfeld R, Ulland TK (2017). A unique microglia type associated with restricting development of Alzheimer’s disease. Cell.

[150] Delizannis AT, Nonneman A, Tsering W, De Bondt A, Van den Wyngaert I, Zhang B (2021). Effects of microglial depletion and TREM2 deficiency on Aβ plaque burden and neuritic plaque tau pathology in 5XFAD mice. Acta Neuropathol Commun.

[151] Rothman SM, Tanis KQ, Gandhi P, Malkov V, Marcus J, Pearson M (2018). Human Alzheimer’s disease gene expression signatures and immune profile in APP mouse models: a discrete transcriptomic view of Aβ plaque pathology. J Neuroinflammation.

[152] McFarland KN, Ceballos C, Rosario A, Ladd T, Moore B, Golde G (2021). Microglia show differential transcriptomic response to Aβ peptide aggregates ex vivo and in vivo. Life Sci Alliance.

[153] Lodder C, Scheyltjens I, Stancu IC, Botella Lucena P, Gutiérrez de Ravé M, Vanherle S (2021). CSF1R inhibition rescues tau pathology and neurodegeneration in an A/T/N model with combined AD pathologies, while preserving plaque associated microglia. Acta Neuropathol Commun.

[154] Natunen T, Martiskainen H, Marttinen M, Gabbouj S, Koivisto H, Kemppainen S (2020). Diabetic phenotype in mouse and humans reduces the number of microglia around β-amyloid plaques. Mol Neurodegener.

[155] Romero-Molina C, Navarro V, Sanchez-Varo R, Jimenez S, Fernandez-Valenzuela JJ, Sanchez-Mico MV (2018). Distinct microglial responses in two transgenic murine models of TAU pathology. Front Cell Neurosci.

[156] Sobue A, Komine O, Hara Y, Endo F, Mizoguchi H, Watanabe S (2021). Microglial gene signature reveals loss of homeostatic microglia associated with neurodegeneration of Alzheimer’s disease. Acta Neuropathol Commun.

[157] Gerrits E, Brouwer N, Kooistra SM, Woodbury ME, Vermeiren Y, Lambourne M (2021). Distinct amyloid-β and tau-associated microglia profiles in Alzheimer’s disease. Acta Neuropathol.

[158] Olah M, Menon V, Habib N, Taga MF, Ma Y, Yung CJ (2020). Single cell RNA sequencing of human microglia uncovers a subset associated with Alzheimer’s disease. Nat Commun.

[159] Xu J, Zhang P, Huang Y, Zhou Y, Hou Y, Bekris LM (2021). Multimodal single-cell/nucleus RNA sequencing data analysis uncovers molecular networks between disease-associated microglia and astrocytes with implications for drug repurposing in Alzheimer’s disease. Genome Res.

[160] Sala Frigerio C, Wolfs L, Fattorelli N, Thrupp N, Voytyuk I, Schmidt I (2019). The major risk factors for Alzheimer’s disease: age, sex, and genes modulate the microglia response to Aβ plaques. Cell Rep.

[161] Sierksma A, Lu A, Mancuso R, Fattorelli N, Thrupp N, Salta E (2020). Novel Alzheimer risk genes determine the microglia response to amyloid-β but not to TAU pathology. EMBO Mol Med.

[162] Marschallinger J, Iram T, Zardeneta M, Lee SE, Lehallier B, Haney MS (2020). Lipid-droplet-accumulating microglia represent a dysfunctional and proinflammatory state in the aging brain. Nat Neurosci.

[163] St-Pierre MK, Carrier M, Lau V, Tremblay MÈ, Jahani-Asl A (2022). Investigating microglial ultrastructural alterations and intimate relationships with neuronal stress, dystrophy, and degeneration in mouse models of Alzheimer’s disease. Neuronal cell death.

