# Supplementary figures and images for: Astrocytes display ultrastructural alterations and heterogeneity in the hippocampus of aged APP-PS1 mice and human post-mortem brain samples
Source: J Neuroinflammation. 2023 Mar 14;20:73. doi: 10.1186/s12974-023-02752-7 (PMC10015698; doi:10.1186/s12974-023-02752-7)

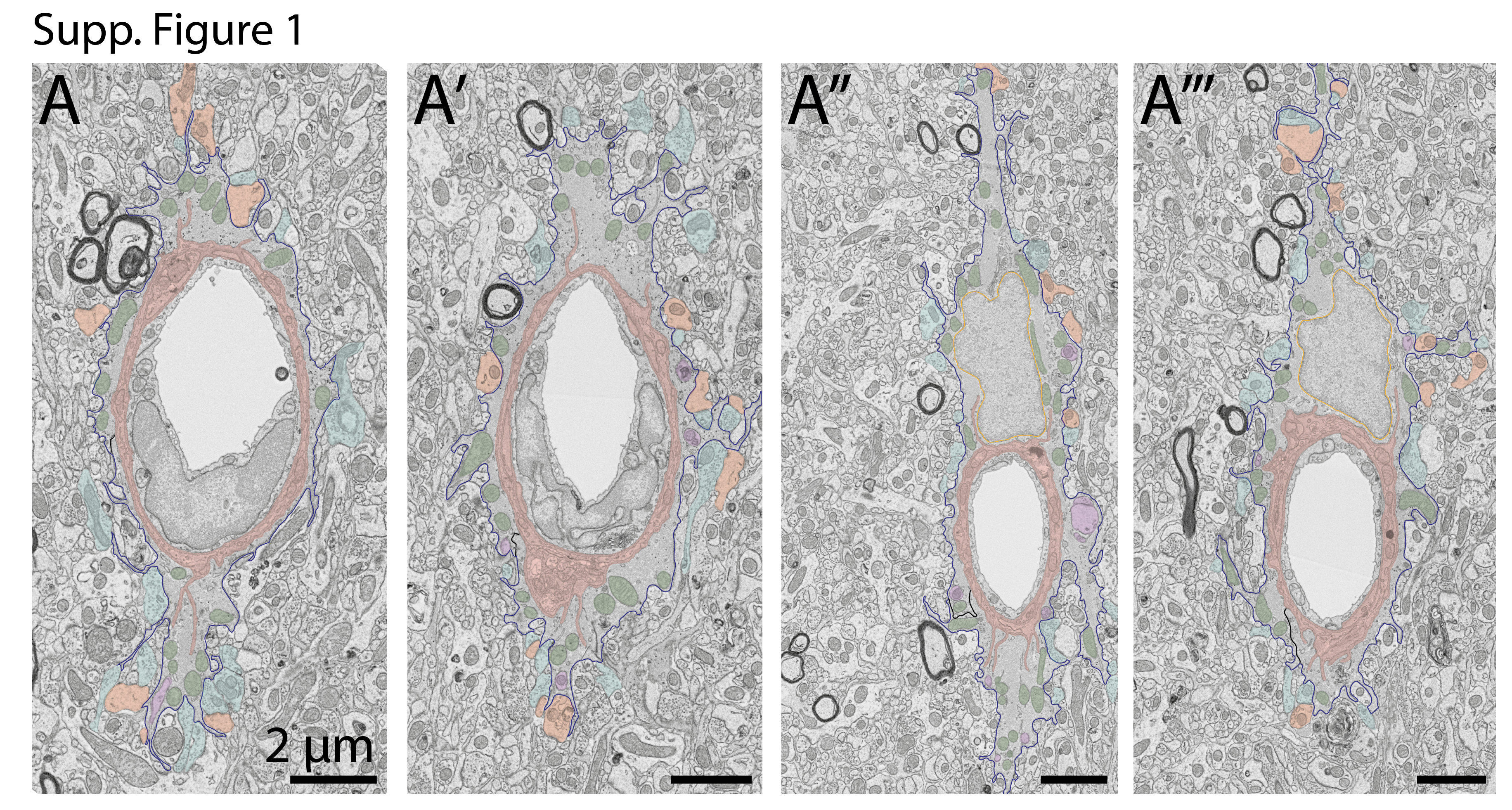

Supplement: Supplementary file 1 — Additional file 1: Figure S1. A blood vessel-associated dark astrocyte imaged serially distances displays several phagosomes. In A–A″, pictures of a dark astrocyte taken serially at a distance of 5–6 µm were acquired. The dark astrocytic cell body, associated with a blood vessel, shows numerous contacts with dendritic spines (pseudo-colored in orange) and axon terminals (pseudo-colored in blue) and contains several partially digested phagosomes (pseudo-colored in purple), notably axon terminals and dendritic spines. Yellow = nuclear membrane, red pseudo-coloring = blood vessel, orange pseudo-coloring = dendritic spine, blue pseudo-coloring = axon terminals, green pseudo-coloring = mitochondria, purple pseudo-coloring = partially digested phagosomes [file 12974_2023_2752_MOESM1_ESM.png]
